# (Inner-Product) Functional Encryption with Updatable Ciphertexts

**DOI:** 10.1007/s00145-023-09486-y

**Published:** 2023-12-15

**Authors:** Valerio Cini, Sebastian Ramacher, Daniel Slamanig, Christoph Striecks, Erkan Tairi

**Affiliations:** 1 NTT Research, Sunnyvale, CA USA; 2 AIT Austrian Institute of Technology, Vienna, Austria; 3 Research Institute CODE, Universität der Bundeswehr München, Munich, Germany; 4 TU Wien, Vienna, Austria

## Abstract

We propose a novel variant of functional encryption which supports ciphertext updates, dubbed ciphertext-updatable functional encryption. Such a feature further broadens the practical applicability of the functional encryption paradigm and allows for fine-grained access control even after a ciphertext is generated. Updating ciphertexts is carried out via so-called update tokens which a dedicated party can use to convert ciphertexts. However, allowing update tokens requires some care for the security definition. Our contribution is threefold:

- We define our new primitive with a security notion in the indistinguishability setting. Within CUFE, functional decryption keys *and* ciphertexts are labeled with tags such that only if the tags of the decryption key and the ciphertext match, then decryption succeeds. Furthermore, we allow ciphertexts to switch their tags to any other tag via update tokens. Such tokens are generated by the holder of the main secret key and can only be used in the desired direction.
- We present a generic construction of CUFE for any functionality as well as predicates different from equality testing on tags which relies on the existence of indistinguishability obfuscation (iO).
- We present a practical construction of CUFE for the inner-product functionality from standard assumptions (i.e., LWE) in the random-oracle model. On the technical level, we build on the recent functional encryption schemes with fine-grained access control and linear operations on encrypted data (Abdalla et al., AC’20) and introduce an additional ciphertext updatability feature. Proving security for such a construction turned out to be non-trivial, particularly when revealing keys for the updated challenge ciphertext is allowed. Overall, such construction enriches the set of known inner-product functional encryption schemes with the additional updatability feature of ciphertexts.

## Introduction

Functional encryption [19, 52, 55] is an exciting encryption paradigm that allows fine-grained access control over encrypted data. In contrast to conventional encryption, which is all-or-nothing, in functional encryption (FE) there is a main secret key $$msk $$ that allows to generate constrained functional decryption keys. More precisely, every decryption key $${sk}_f$$ is associated with a function *f* and given an encryption $$\textsf{Enc} (mpk,x)$$ of some message *x* under the main public key $$mpk $$, the decryption with $${sk}_f$$ only reveals *f*(*x*), but nothing more about *x*.^1^

Since its introduction, FE has been subject to intense study which can broadly be categorized into two areas. Firstly, works that consider general functionalities and thereby mostly focusing on feasibility results. This typically results in constructions beyond practical interest, as they rely on indistinguishability obfuscation (iO) or need to impose severe restrictions on the number of keys given to an adversary. Secondly, works that restrict the power by only supporting limited classes of functions that are of particular interest for practical applications, i.e., linear and quadratic functions. Here, the main focus is then on concrete and efficient constructions. One such approach that attracted a lot of research are FE schemes for the inner-product functionality (IPFE), i.e., keys are associated with vectors $\vec {y}$, messages are vectors $\vec {x}$ and decryption reveals $\langle \vec {x}, \vec {y}\rangle$. Initially proposed by Abdalla et al. [2], a line of work improved the security guarantees [3, 13, 15, 16, 24], extended it to the multi-input [8, 12] as well as the decentralized setting [4–6, 22, 46]. Although this functionality is very simple, it has already shown to be useful in privacy-preserving machine learning [49], money-laundering detection [31], search in encrypted data streams [18], video data analytics,^2^ or data marketplaces [43].

*Limitations of large-scale deployment of FE* A problem for the practical adoption of FE is that every issued functional decryption key inherently leaks some information. For the inner-product functionality and thus IPFE, this is particularly problematic. Specifically, if *n* is the dimension of the vectors, then obtaining *n* decryption keys in general allows to recover the full plaintext. Consequently, as soon as IPFE is deployed in some larger-scale setting, this represents a severe limitation. To mitigate this problem and make IPFE more practical, Abdalla, Catalano, Gay, and Ursu [9] recently introduced the notion of IPFE with fine-grained access control providing strong security guarantees.^3^ Loosely speaking, the idea is that ciphertexts are produced with respect to an access policy (e.g., expressed by monotone span programs) and decryption keys are in addition to being bound to a function also associated with an attribute. Decryption then only works if the attribute in the key satisfies the access policy in the ciphertext. It is important to stress that when aiming for reasonable security which allows collusion of functional decryption keys, this approach is non-trivial as a naive composition of IPFE with attribute-based encryption (ABE) or identity-based encryption (IBE) suffers from simple mix-and-match attacks. Abdalla et al. provide pairing-based attribute-based constructions covering monotone span programs (AB-IPFE) and lattice-based identity-based constructions (IB-IPFE). Nguyen et al. [51] propose more efficient pairing-based constructions and investigate the approach of Abdalla et al. in a multi-client setting. Recently, Lai et al. [45] as well as Pal and Dutta [53] also present lattice-based AB-IPFE constructions.

This concept of Abdalla et al. firstly mitigates the leakage problem of plain IPFE, as now this inherent limitation on the number of issued functional decryption key only applies per identity in IB-IPFE (or attribute policy in AB-IPFE). This can be viewed as partitioning the keys such that the aforementioned limitation applies to each of these partitions, making it much more scalable. Secondly, it more closely reflects the situation in large-scale systems where even in the case of FE, one wants to enforce a more fine-grained control over who is allowed to learn some particular information of the encrypted plaintexts. Thirdly, this concept overcomes the problem of a trivial approach, i.e., encrypting data separately under an IPFE public key for each recipient, which would result in a linear blow-up of the ciphertexts.

*Motivation towards more flexibility in fine-grained access control* Abdalla et al. [9] make an important step towards applicability of FE in large-scale systems. But it still seems limited when it comes to dynamic aspects. For instance, the medical example used in [9] envisions that doctors in a hospital may be able to compute on a different set of encrypted data than employees of a health insurance company. What happens if the access to data for the insurance company should be expanded? This would either mean to encrypt *all* the data anew under the policy that is satisfied by the insurance company or to issue additional keys to the insurance company. While in this medical setting this might still be manageable, there are other examples where this seems hard to achieve.

Let us therefore consider the emerging domain of data marketplaces.^4^ These are platforms that allow customers to buy access to data or statistical analysis on data offered by a potentially huge set of data owners via data brokers. The available data sets can range from business intelligence and research, demographic or health, firmographic, and market data to public data. (IP)FE seems to be an interesting tool for this application. But while the use of IPFE (in a multi-client setting) has recently been proposed in [43] to realize a privacy-aware data marketplace, it does so in a way that reveals the evaluations in plain to the data brokers. Now, one could imagine using the approach in [9] to let data owners encrypt their data under certain policies (or identities), whereas data buyers are given functional keys (with respect to a certain identity or attribute) and data brokers basically only distribute the data (and possibly perform some aggregation tasks). Still, it seems cumbersome to have a fine-grained control over what buyers can access if the access policies are fixed in the ciphertexts.

We now envision that in addition to having a fine-grained control, we allow the data brokers to update the policies (attributes/identities) in existing ciphertexts in order to add more flexibility. Let us now focus on the specific case of policies being represented via the equality predicate, and thus ciphertexts and function keys are labeled and decryption yields the function of the message if both labels match. We call those labels *tags* and one can also think of these labels as identities (as done in [9]). Data brokers should have the capability to update ciphertexts in a way that they can change the tags in ciphertexts using some additional information (called an update token), but they should not learn the function evaluations and thus the privacy of the data of the owners is guaranteed. To keep a fine-grained control over ciphertext updates in such a broker scenario, we want to restrict the updates of a ciphertext to a single update and the token to only work in one direction, i.e., from tag *t* to $t'$ but not vice versa. Thus, already updated ciphertexts cannot be updated anymore. While it is possible to consider schemes that support multiple updates and/or bidirectional tokens, we believe that this is rather dangerous in such applications. For instance, this could allow moving ciphertexts to tags for which they were not intended, e.g., from a tag *t* to $t'$ and then to $t''$ via two updates, whereas it might be not intended that it is possible to move all ciphertexts from *t* to $t''$, but rather only ones under *t* to $t'$ and ones under $t'$ to $t''$.

We note that this functionality goes beyond what is provided by IPFE with fine-grained access control due to Abdalla et al. [9], as in their work ciphertexts are not updatable, i.e., they do not straightforwardly provide the possibilities that a tag (identity) in a ciphertext can be changed. But as we will see, the work in [9] can serve as a starting point for our lattice-based construction. We note that a trivial construction based upon [9] that encrypts a message multiple times under different tags (identities) in parallel fails to provide the desired functionality. In particular, it does not allow to dynamically decide to which tag a ciphertext can be updated as the desired tags would have to be known at the time of producing the ciphertext, something that we want to avoid in our approach to solve the above problem! Consequently, we are looking for a solution where we can potentially switch a ciphertext to any tag from a large (i.e., exponential) tag space.

Since currently (IP)FE schemes that achieve the desired properties are absent in the cryptographic literature, in this work we ask:

*Can we define and construct (IP)FE schemes with fine-grained access control and ciphertext updatability?*

### Our Contribution

We answer the above question affirmatively via our threefold contribution

- We define a new primitive dubbed ciphertext-updatable functional encryption (CUFE) along with a security notion in the indistinguishability setting. Within CUFE, functional decryption keys *and* ciphertexts are labeled with tags such that only if the tag in the decryption key and ciphertext match, then decryption succeeds. Furthermore, we allow fresh ciphertexts to update its tag $$t $$ to any other tag $$t '$$ via so-called update tokens. An update token from $$t $$ to $$t '$$ is generated by the holder of the main secret and can only be used in the desired direction, i.e., from $$t $$ to $$t '$$. In a nutshell, the distinguishing feature is that we allow changing the tag *after* a ciphertext was generated (which is not known to be achieved by existing work).
- We present a generic construction of CUFE for any functionality and more powerful predicates than equality testing on tags, which relies on the existence of indistinguishability obfuscation (iO).
- We present a practical construction of CUFE for the inner-product functionality from standard assumptions (i.e., the learning-with-errors (LWE) assumption) in the random-oracle model. Proving security for such a construction turned out to be non-trivial, particularly when revealing keys for the updated challenge ciphertext is allowed. In general, this further enriches the approach presented in line of Abdalla et al. [9] with the updatability feature of ciphertexts. Notably, our construction relies on lattice-based assumptions which are plausibly post-quantum.

*Defining ciphertext updatability for FE* CUFE can be seen as tag-based FE scheme with tag space $$\mathcal {T} $$. As in FE, key generation outputs a main public-secret key pair $$(mpk,msk)$$, where the decryption keys $$sk _{f,t}$$ for some function $$f \in \mathcal {F} $$ and tag $$t \in \mathcal {T} $$ are derived from $$msk $$. In CUFE, however, $$msk $$ is also used to derive update tokens $$\Delta _{t \rightarrow t '}$$. Now, encryption takes some tag $$t $$ and message $$x $$ and outputs a ciphertext $$C _t $$. Then, using $$\Delta _{t \rightarrow t '}$$, any honest-but-curious party^5^ can take the update token to update $$C _t $$ to $$C _{t '}$$ without learning anything about the encrypted message. Correctness guarantees that if the tags of the function key and the ciphertext match, and only a single update has happened, then decryption succeeds and outputs $$f (x)$$.

Defining security needs some care as we want that tokens can update ciphertexts only toward the tag specified in the update token and updated ciphertext should not be allowed to be further updated. That is, a token $$\Delta _{t \rightarrow t '}$$ can only switch tags from $$t $$ to $$t '$$ and not vice versa. As in the work of Abdalla et al. [9], the adversary is allowed to query decryption keys for any functionality $$f $$ such that the function evaluation on the challenge ciphertext yields $$f (x_0)=f (x_1)$$, for adversarially chosen messages $$x_0,x_1$$, if the policy is fulfilled. In our constructions, we restrict the policy to the equality test on tags of the functional decryption key and the ciphertext (we discuss extensions in Sect. 4.3) which ensures a simple access control for our envisioned applications.

Concerning updated ciphertexts, we have the following situation. Since the concept of update tokens is not foreseen in conventional forms of FE, we need to consider additional aspects for our security notions. We have to deal with the fact that tokens can potentially not only be used to update ciphertexts from some tag $$t $$ to another tag $$t '$$, but could also be used to invert a ciphertext update. This is partly reminiscent of providing adequate and strong security guarantees in proxy re-encryption (PRE) [26, 28]. Having those in mind, we define an indistinguishability-based notion $$\mathsf {IND\text {-}CUFE\text {-}CPA}$$, which guarantees that an adversary cannot distinguish ciphertexts for a certain challenge target tag and adversarially chosen messages.

More concretely, as outlined in our motivation, we only want to allow updating the tags of ciphertexts *once* and only in *one direction*. In order to capture these properties, we provide the adversary in addition to a key generation oracle (as in plain FE) access to additional oracles. Firstly, we allow the adversary to adaptively query corrupted and honest update tokens as well as also provide encryption and honest-ciphertext-update oracles. Furthermore, we want to naturally allow the adversary to see decryption keys for honestly updated challenge ciphertexts.

We show that we can prove our CUFE construction from LWE secure in such a model for the inner-product functionality. Indeed, the tricky part in the proof is to allow the adversary to retrieve functional decryption keys for honestly updated challenge ciphertexts (i.e., it does not see the update token, but has access to an update oracle; see below for detailed discussion). We note that our iO-based construction satisfies the security model for any functionality (see below).

*CUFE for any function from iO.* The starting point of our construction is the (semi-adaptively secure) FE construction due to Waters [57], which relies on indistinguishability obfuscation (iO) and the punctured programming approach. The main ingredient of Waters’ construction is a primitive called puncturable deterministic encryption (PDE), which can be constructed from puncturable PRFs using the hidden trigger mechanism of Sahai and Waters [56]. A PDE scheme is a symmetric and deterministic encryption scheme, which additionally has a feature that given a key $k_{\textsf{pde}}$ and a pair of messages $m_0,m_1$, it produces a punctured key $k_{\textsf{pde}}^{m_0,m_1}$ that can decrypt all ciphertexts except for those encrypting either $m_0$ or $m_1$.^6^ Using PDE one can construct a (semi-adaptively secure) FE scheme as follows: The setup algorithm samples a puncturable PRF key $k_{\textsf{prf}}$ for function *F*, which it sets as the main secret key, and generates an obfuscation of the program PInit, which it sets as the main public key. The program PInit takes as input a randomness *r*, computes a point $p = \textsf{PRG} (r)$, derives a PDE key as $k_{\textsf{pde}} = F(k_{\textsf{prf}},p)$, and outputs the pair $(p, k_{\textsf{pde}})$. The encryption algorithm can then use the obfuscated program PInit to encrypt a message *m* by first sampling a randomness *r*, running the obfuscated program on *r* to receive $(p, k_{\textsf{pde}})$, and finally, computing the ciphertext as $C:= (p, c:= \textsf{Enc} _{\textsf{pde}}(k_{\textsf{pde}}, m))$. The functional secret key ${sk}_f$, for a function *f*, is also created as an obfuscation of a program PKey, which has *f* hardcoded. This program takes as input a ciphertext $C:= (p, c)$, uses *p* to derive the key $k_{\textsf{pde}}$, decrypts *c* using $k_{\textsf{pde}}$ to obtain the message *m*, and finally, outputs *f*(*m*). Hence, the decryption algorithm simply involves running the obfuscated program PKey on the ciphertext.

In order to introduce tags for the ciphertexts, a first step is to extend the PDE to its tag-based variant that we dubbed puncturable tag-based deterministic encryption (PTDE). It works analogously to PDE, except that the ciphertexts are associated with tags and puncturing happens not only at a pair of messages $m_0,m_1$, but also at a tag *t*. Hence, a punctured key $k_{\textsf{ptde}}^{t,m_0,m_1}$ can decrypt all ciphertexts except for those encrypting either $m_0$ or $m_1$ under the tag *t*. Now, the challenging part is to update the ciphertexts. In order to restrict that an updated ciphertext cannot be updated anymore, we use two different puncturable PRF keys as part of the main secret key, $k_{\textsf{prf},o}$ for the original ciphertexts and $k_{\textsf{prf},u}$ for the updated ciphertext. Analogous to the aforedescribed construction of Waters [57], these PRF keys are used to derive PTDE keys in our case. For the update operation, we now need to switch the ciphertexts encrypted under the key $k_{\textsf{ptde}}$ (derived from $k_{\textsf{prf},o}$) and tag *t* to a new ciphertext under the key $k_{\textsf{ptde}}'$ (derived from $k_{\textsf{prf},u}$) and tag $t'$. In order to do this we introduce a third program, called PUpdate, which given as input a ciphertext $C_t$ (under a tag *t*) and a randomness *r*, first decrypts the input ciphertext $C_t$, and then, re-encrypts it deterministically under the new key $k_{\textsf{ptde}}'$ and tag $t'$ to produce the updated ciphertext $C_t'$. Due to the deterministic nature of the used cryptographic primitives, such as PTDE and puncturable PRF, we can rely solely on (plain) iO for the update operation, instead of requiring probabilistic iO [25].

*CUFE for inner-products from standard assumptions* The starting point for the construction from standard assumptions is the identity-based inner-product functional encryption scheme from the LWE assumption by Abdalla et al. [9]. Their construction essentially combines the LWE-based inner-product FE scheme from Agrawal et al. [15]—we will refer to this scheme as ALS—with a LWE-based IBE scheme, e.g., the IBEs from [37] or [1]. The latter is especially of interest for us: Starting from a public key $\textbf{A}$, it is possible to derive an identity-specific matrix $\textbf{A}_{id}$ for some identity *id*. This $\textbf{A}_{id}$ describes a trapdoor function for which it is hard to compute a short preimage. Yet, given the trapdoor for $\textbf{A}$, which is stored as part of the main secret key, it is possible to derive $sk _{id}$ as trapdoor for $\textbf{A}_{id}$. Notably, $sk _{id}$ is a matrix which can be projected to functional decryption keys for inner-products $\langle \cdot , \vec {y} \rangle$, hence giving $sk _{id,\vec {y}}$.

While this idea incidentally gives rise to a tag-based inner-product FE construction, producing update tokens to transform ciphertexts from the source to the target tag is non-obvious. We want to note, however, that this is one of the core challenges that is solved by proxy re-encryption in the public-key encryption setting. It is, however, non-trivial to combine a proxy re-encryption scheme with a functional encryption scheme without running into issues with collusion. Indeed, consider a black-box approach that combines both worlds by encrypting the FE ciphertext with a PRE. Now, consider two colluding users *t* and $t'$ who have functional secret keys for distinct *f* and $f'$. Now, if a ciphertext is re-encrypted to *t*, they can use their PRE secret key to remove the PRE layer. Then, both *t* and $t'$ can evaluate their functions by simply sharing the decapsulated FE ciphertext. Therefore, a CUFE scheme requires tighter intertwining of the two concepts to prevent mix-and-match-style and other attacks.

Still, ideas found in lattice-based proxy re-encryption constructions help us to turn ALS combined with tag-based keys into a secure CUFE. We quickly revisit the construction by Fan and Liu [33] of a tag-based proxy re-encryption scheme. Their idea is to set up the user-specific matrices from a global public matrix $\textbf{A}$. Given such a fixed matrix $\textbf{A}$, the matrix for a user *u* is then set to be $\textbf{A}_u = [ \textbf{A} | \textbf{A}_{u,1} |\textbf{A}_{u,2} ]$ where $\textbf{A}_{u,i} = -\textbf{AR}_{u,i}$ with $\textbf{R}_{u,i}$, for $i = 1, 2$ contained in the secret key. Encryption follows a dual-Regev approach [37] based on the user dependent matrix $\textbf{A}_u$ and a random freshly sampled tag $t\in \mathcal {T}$. Re-encryption keys from user *u* to user $u'$ are generated by sampling matrices $\textbf{X}_{01}, \textbf{X}_{02}, \textbf{X}_{11}, \textbf{X}_{12}$ using $\textbf{R}_{u,1}, \textbf{R}_{u,2}$ such that

$$\begin{aligned}{}[\textbf{A} | -\textbf{A}_{u,1} + h(1) \textbf{G} | -\textbf{A}_{u,2} + \textbf{B}] \begin{bmatrix} \textbf{I} &{} \textbf{X}_{0,1} &{} \textbf{X}_{0,2} \\ 0 &{} \textbf{X}_{1,1} &{} \textbf{X}_{1,2} \\ 0 &{} 0 &{} \textbf{I} \\ \end{bmatrix} = [\textbf{A} | -\textbf{A}_{u',1} + h(2) \textbf{G} | -\textbf{A}_{u',2} + \textbf{B}] \end{aligned}$$

for any matrix $\textbf{B}$. In their construction, *h* is a map used to describe the “ciphertext level” (either freshly generated, *h*(1) or updated, *h*(2)), whereas $\textbf{B}$ stems from a function producing matrices on input of a tag and the map *h*. Using as tag space $\mathcal {T}$ a large set with “unit differences” property, as introduced in [47], i.e., for any for any $t_i,t_j\in \mathcal {T}$, $t_i\ne t_j$, one has $h(t_i - t_j) = h(t_i) - h(t_j )\in \mathbb {Z}_q^{n\times n}$ is invertible, Fan and Liu prove their construction secure in the standard model. Their proof strategy crucially relies on the “unit differences” property together with the fact that the scheme is tag-based: The “challenge” tag, i.e., the tag associated with the challenge ciphertext, is randomly sampled at the beginning of the security game, and the public parameter is produced by embedding such “challenge” tag in them. This allows the reduction to correctly answer any allowed adversary’s query, while at the same time embedding an LWE instance in the challenge ciphertext.

The setting of CUFE is, however, vastly different in nature as ciphertexts are not equipped with levels, there are no per-user public keys, and tags have a different meaning, and in particular, they are not randomly sampled at encryption time, but are specified by the encryptor. Yet, this method to set up the matrices such that one can update dual-Regev style ciphertexts from one matrix to another is helpful to construct the update tokens. Additionally, with dual-Regev inspired ciphertexts we are also able to set up keys as matrices in such a way that we are able to first sample a tag-specific trapdoor from the main secret key which is then projected to a functional secret key. Consequently, our construction intertwines the functional encryption features from ALS with tag-based ciphertext updates in a non-black-box manner.

As the construction is not black-box, neither is the proof. First, we move to the random-oracle model in order to embed the challenge tag in the public parameters, even though in our setting such tag is specified by the encryptor, by crucially exploiting the fact that the reduction can guess the challenge tag among the random-oracle queries made by the adversary. Given such modification, the main technical challenge in the proof comes from having to produce updates of the challenge ciphertext and function keys for the respective target tags. Embedding an ALS instance (as done for the challenge identity in [9]) for each of these tags does not work as the different instances should be related in order to simulate the derived matrices of these tags correctly. On the other hand, using a single ALS instance to simulate function keys for multiple tags leads, if done in the trivial way, to producing function keys related to each other, and thus again to a view for the adversary distinguishable from the expected one. However, this drawback can be overcome by “re-randomizing” the function keys in a way that it “hides” the function key provided by the ALS challenger (similarly to Lai et al. [45]). In this way, the adversary’s view is indistinguishable from that in the real experiment. We remark that since the reduction needs to perform guesses in order to correctly produce public parameters and answer adversary’s queries, one has to make sure that the probability space over which the reduction needs to guess has at most polynomial size. In particular, such constraint will allow us to prove the lattice-based scheme secure, but only against adversary that can request at most a bounded number of update tokens per tag and honest updates of the challenge ciphertext. We discuss more in detail these restrictions in Sect. 5. On the other hand, while this is certainly a limitation in general, for the concrete applications we envision, one can always set parameters so that such bounds are large enough to accommodate requirements of real world scenarios.

### Related Work

While we are not aware of any previous work that tries to achieve the desired goals via ciphertext updatability, a related concept is that of controlled functional encryption (C-FE) [50]. This approach enhances FE with an authority that needs to be involved in the decryption process and thus allows a fine-grained control over which ciphertexts can be decrypted by a holder of a functional key. Consequently, the access control is enforced by the authority and by dynamically changing which user is allowed to decrypt which ciphertexts one can view this as achieving similar goals as with ciphertext updatability. However, the major difference is that C-FE requires an interactive decryption procedure between the user and authority and thus requires the authority to be online and available all the time. This would potentially hinder scalability in large-scale systems. In contrast, our approach is oblivious to the users. Furthermore, the requirement of an always online authority that needs to be fully trusted might be problematic and undesirable. This trust issue has recently been addressed by distributing the trust in the authority via the concept of Multi-Authority C-FE [11], however, this incurs further communication overhead. Another related (but conceptually different) line of work is updating policies in ABE [34, 42]. In general, these works combine ciphertext-policy ABE with PRE in order to update the policy associated with the ciphertext. However, these works neither consider (IP)FE schemes nor are sufficient for our envisioned applications. Our work can be seen as a combination of IBE/ABE with FE augmented by updatability, and, hence, updatability needs to consider and tie both parts together.

## Preliminaries

**Notation** For $n\in \mathbb {N}$, let $[n]:=\{1,\ldots ,n\}$, and let $\lambda \in \mathbb {N}$ be the security parameter. For a finite set $\mathcal {S}$, we denote by $s\leftarrow \mathcal {S}$ the process of sampling $s$ uniformly from $\mathcal {S}$. Let $y\leftarrow A(\lambda ,x)$ be the process of running an algorithm $A$ on input $(\lambda ,x)$ with access to uniformly random coins and assigning the result to $y$. (We may omit to mention the $\lambda$-input explicitly and assume that all algorithms take $\lambda$ as input.) To make the random coins $r$ explicit, we write $A(\lambda ,x;r)$. We use $\bot$ to indicate that an algorithm terminates with an error and $A^B$ when *A* has oracle access to *B*, where *B* may return $\top$ as a distinguished special symbol. We say an algorithm $A$ is probabilistic polynomial time (PPT) if the running time of $A$ is polynomial in $\lambda$. Given $\vec {x}\in \mathbb {Z}^n$, we denote by $\Vert \vec {x}\Vert$ its Euclidean norm, i.e., for $\vec {x}=(x_i)_{i \in [n]}$, we have $\Vert \vec {x}\Vert :=\sqrt{\sum _{i=1}^n x_i^2}$. For a matrix $\textbf{R}$, by $\widetilde{\textbf{R}}$, we denote the result of applying Gram–Schmidt orthogonalization to the columns of $\textbf{R}$. By $\Vert \textbf{R}\Vert$, we will denote the Euclidean norm of the longest column of $\textbf{R}$, and by $s_1(\textbf{R})$ its spectral norm, i.e., the largest singular value of $\textbf{R}$. A function $f$ is negligible if its absolute value is smaller than the inverse of any polynomial (i.e., if $\forall c\exists k_0\forall \lambda \ge k_0:|f(\lambda )|<1/ \lambda ^c$). We may write $q=q(\lambda )$ if we mean that the value $q$ depends polynomially on $\lambda$. Given two different distributions *X* and *Y* over a countable domain *D*, we denote their statistical distance as ${\textsf{SD}}(X,Y)=\frac{1}{2}\sum _{d\in D}|X(d)-Y(d)|$ and say that *X* and *Y* are ${\textsf{SD}}(X,Y)$ close.

### Pseudorandom Generators

We recall the definition of a (Boolean) pseudorandom generator (PRG).

#### Definition 1

(Pseudorandom Generator) A stretch-$$m(\cdot )$$ pseudorandom generator is a (Boolean) function $$\textsf{PRG} :\{0,1\}^* \rightarrow \{0,1\}^*$$ mapping *n*-bit inputs to *m*(*n*)-bit outputs (also known as the stretch) that is computable by a uniform PPT machine, and for any non-uniform PPT adversary $$A $$, there exists a negligible function $$\textsf{negl}$$, such that, for all $$n \in \mathbb {N} $$, the following holds

$$\begin{aligned} \left| \Pr _{r \leftarrow \{0,1\}^n}\left[ \,A (\textsf{PRG} (r)) = 1\,\right] - \Pr _{z \leftarrow \{0,1\}^m}\left[ \,A (z) = 1\,\right] \right| = \textsf{negl}(\lambda ). \end{aligned}$$

### Puncturable Pseudorandom Functions

Puncturable pseudorandom functions (PRFs), introduced by Sahai and Waters [56], are PRFs for which a key can be given out, such that it allows evaluation of the PRF on all inputs, except for a designated polynomial-size set of inputs.

#### Definition 2

(Puncturable PRFs [56]) A puncturable family of PRFs $\textsf{PRF}$ is given by a triple of algorithms $(\textsf{Gen}, F, \textsf{Puncture})$ and a pair of computable functions $n = n(\lambda )$ and $m = m(\lambda )$, satisfying the following conditions:

*Functionality preserved under puncturing* For every PPT adversary $$A $$ that outputs a set $$S \subseteq \{0,1\}^{n}$$, such that for all $$x \in \{0,1\}^{n}$$ where $$x \not \in S$$, we have that:

$$\begin{aligned} \Pr \left[ {F(k, x) = F(k^S, x) \mid k \leftarrow \textsf{PRF}.\textsf{Gen} _F(1^\lambda ), k^{S} \leftarrow \textsf{PRF}.\textsf{Puncture}_{F}(k,S)}\right] = 1. \end{aligned}$$

*Pseudorandom at punctured points.* For every PPT adversary $(A _1,A _2)$, where $A _1$ outputs a set $S \subseteq \{0,1\}^{n}$ and a state $\sigma$, consider an experiment that samples $k \leftarrow \textsf{PRF}.\textsf{Gen} _F(1^\lambda )$ and $k^S \leftarrow \textsf{PRF}.\textsf{Puncture}_F(k,S)$, then we have

$$\begin{aligned} \left| \Pr \left[ {A _2(\sigma , k^S, S, F(k, S)) = 1}\right] - \Pr \left[ {A _2(\sigma , k^S, S, U_{m\cdot |S|}) = 1}\right] \right| = \textsf{negl}(\lambda ), \end{aligned}$$

where *F*(*k*, *S*) denotes the concatenation of $F(k,x_1),\ldots ,F(k,x_k)$, such that $S = \{x_1,\ldots ,x_k\}$ is the enumeration of the elements of *S* in lexicographic order and $U_\ell$ denotes the uniform distribution over $\ell$ bits.

The GGM tree-based PRF construction [36] from one-way functions yields a puncturable PRF where the punctured key sizes are polynomial in the size of the set *S* [20].

In this work, we also make use of *injective* families of PRFs [56, 57]:

#### Definition 3

A statistically injective (puncturable) PRF family with failure probability $\epsilon (\cdot )$ is a family of (puncturable) PRFs $\textsf{PRF}$, such that with probability $1 - \epsilon (\lambda )$ over the random choice of key $k \leftarrow \textsf{PRF}.\textsf{Gen} _F(1^\lambda )$, we have that $F(k,\cdot )$ is injective.

If the failure probability function $\epsilon (\cdot )$ is not specified, then we assume that $\epsilon (\cdot )$ is a negligible function in the security parameter $\lambda$. Sahai and Waters [56] showed that assuming the existence of one-way functions there exists statistically injective puncturable PRF family with failure probability $2^{-\epsilon (\lambda )}$.

### Indistinguishability Obfuscation

We recall the definition of indistinguishability obfuscation.

#### Definition 4

(Indistinguishability Obfuscator [35]) A PPT algorithm $i\mathcal {O}$ is an indistinguishability obfuscator (iO) for a circuit class $\{\mathcal {C} _\lambda \}_{\lambda \in \mathbb {N}}$ if it satisfies the following conditions:

*Functionality* For any security parameter $\lambda \in \mathbb {N}$, any circuit $C \in \mathcal {C} _\lambda$, and any input *x*, we have that

$$\begin{aligned} \Pr \left[ {C'(x) = C(x) \mid C' \leftarrow i\mathcal {O}(C)}\right] = 1. \end{aligned}$$

*Indistinguishability* For any PPT distinguisher $\mathcal {D}$ and for any pair of circuits $C_0,C_1 \in \mathcal {C} _\lambda$, such that for any input *x*, $C_0(x) = C_1(x)$ and $|C_0|=|C_1|$, it holds that

$$\begin{aligned} | \Pr \left[ {\mathcal {D} (i\mathcal {O}(C_0)) = 1}\right] - \Pr \left[ {\mathcal {D} (i\mathcal {O}(C_1)) = 1}\right] | \le \textsf{negl}(\lambda ). \end{aligned}$$

We further say that $i\mathcal {O}$ is subexponentially secure if for any PPT $\mathcal {D}$ the above advantage is smaller than $2^{-\lambda ^\varepsilon }$ for some $0< \varepsilon < 1$.

## Ciphertext-Updatable Functional Encryption

We present our definitional framework of ciphertext-updatable functional encryption (CUFE). CUFE is a tag-based functional encryption (FE) scheme defined on functionality $$\mathcal {F} :\mathcal {X} \rightarrow \mathcal {Y} $$ and tag space $$\mathcal {T} $$. Key generation outputs a main public-secret key pair $$(mpk,msk)$$, where from $$msk $$, the function keys $$sk _{f,t}$$ for some function $$f \in \mathcal {F} $$ and tag $$t \in \mathcal {T} $$ can be derived. Encryption is done according to some tag $$t \in \mathcal {T} $$ and message $$x \in \mathcal {X} $$. Now, if the tag of the function key and the ciphertext match, then decryption succeeds and outputs $$f (x)$$. Furthermore, we want to allow switching of tags, i.e., from $$t $$ to $$t '$$, in a ciphertext once, which is carried out via tokens $$\Delta _{t \rightarrow t '}$$. Such a token can be used to update a ciphertext $$C _t $$ to a ciphertext $$C _{t '}$$ under the tag $$t '$$ specified in the token but not vice versa, i.e., from $$t '$$ to $$t $$.

### Definition 5

A CUFE scheme $\textsf{CUFE}$ for functionality $\mathcal {F} :\mathcal {X} \rightarrow \mathcal {Y}$ with message space $\mathcal {X}$ and tag space $\mathcal {T}$ is a tuple of the PPT algorithms:

$$\textsf{Setup} (\lambda ,\mathcal {F}):$$
on input security parameter $\lambda \in \mathbb {N}$ and a class of functions $\mathcal {F}$, the setup algorithm outputs a main public-secret key pair $(mpk,msk)$.
$$\textsf{KeyGen} (msk,f,t):$$
on input $$msk $$, function $$f \in \mathcal {F} $$, and tag $$t \in \mathcal {T} $$, the key generation algorithm outputs a function key $$sk _{f,t}$$.
$$\textsf{TokGen} (msk,t,t '):$$
on input $$msk $$ and tags $$t,t '\in \mathcal {T} $$, the token generation algorithm outputs an update token $$\Delta _{t \rightarrow t '}$$.
$$\textsf{Enc} (mpk,x,t):$$
on input $$mpk $$, message $$x \in \mathcal {X} $$, and tag $$t \in \mathcal {T} $$, the encryption algorithm outputs a ciphertext $$C _{t}$$ for $$x $$.
$$\textsf{Update} (\Delta _{t \rightarrow t '},C _t):$$
on input an update token $\Delta _{t \rightarrow t '}$ and ciphertext $C _{t}$, the update algorithm outputs an updated ciphertext $UC _{t '}$ or $\bot$.
$$\textsf{Dec} (sk _{f,t '},C _{t}/UC _{t})^{11}:$$
7 on input function key $$sk _{f,t '}$$ and a ciphertext (either a non-updated one $$C _{t}$$ or an updated one $$UC _{t}$$), the decryption algorithm outputs $$f (x) \in \mathcal {Y} $$ if $$t '=t $$, else outputs $$\bot $$.

*Correctness for CUFE* Correctness essentially guarantees that if the tag in a function key and in an (updated) ciphertext match, then decryption succeeds.

More concretely, a CUFE scheme $\textsf{CUFE}$ is correct if for all $\lambda \in \mathbb {N}$, for any $\mathcal {F} :\mathcal {X} \rightarrow \mathcal {Y}$, for any $(mpk,msk)\leftarrow \textsf{Setup} (\lambda ,\mathcal {F})$, for any $f \in \mathcal {F}$, for any $t \in \mathcal {T}$, for any $sk _{f,t}\leftarrow \textsf{KeyGen} (msk,f,t)$, for any $x \in \mathcal {X}$, for any $C _t \leftarrow \textsf{Enc} (mpk,x,t)$, we have that $\textsf{Dec} (sk _{f,t},C _{t}) = f (x)$ holds, and for any $t '\in \mathcal {T} \setminus \{t\}$, for any $\Delta _{t \rightarrow t '}\leftarrow \textsf{TokGen} (msk,t,t ')$, for any $UC _{t '}\leftarrow \textsf{Update} (\Delta _{t \rightarrow t '},C _t)$, we have that $\textsf{Dec} (sk _{f,t '},UC _{t '})=f (x)$ holds.

### Remark 1

Notice that the correctness of the CUFE scheme only guarantees that non-updated ciphertexts for tag $$t $$ can be updated to tag $$t '$$ using the update token $$\Delta _{t \rightarrow t '}$$ and still be decrypted correctly. Looking ahead to the CPA security notion, this will be the only possible use of the update token. Any other successful use (e.g., updating ciphertexts in the reverse direction or updating already updated ciphertexts) will allow the adversary to win the security experiment (see below). Hence, a secure CUFE construction implies that the update token can *only* be used to update a non-updated ciphertext to an updated one (assuming the tags match), but not vice versa and not multiple times (i.e., to “update” an already updated ciphertext is not possible as this would penalize CUFE security).

*Intuition of our CPA security notions for CUFE* Updating ciphertexts via tokens is closely related to the realm of proxy re-encryption (PRE) [10, 17] and, indeed, we start from the recent PRE state-of-the-art security model by Cohen [26] and carefully adapt such a model to our needs in the chosen-plaintext-attack indistinguishability setting. Moreover, due to the updatability of ciphertexts and thus the concept of update tokens not being present in plain FE, we need to require additional aspects for our security guarantees. Such tokens could potentially be used to also switch function keys or even invert updated ciphertexts. In that vein, we define an indistinguishability-based notion, we dub $\mathsf {IND\text {-}CUFE\text {-}CPA}$, which guarantees that an adversary cannot distinguish ciphertexts for a certain target tag $t^*$ and adversarially chosen messages $(x_0^*,x_1^*)$.

We only want to allow updating the tags of ciphertexts via the token, only in one direction, and only from non-updated to updated ciphertexts. In order to capture these properties, we provide the adversary in addition to $\textsf{KeyGen}$ (as in plain FE) access to four more oracles. Two of those additional oracles are related to the generation of tokens and the other two are needed to ensure security related to updatability of honestly generated ciphertexts.

Concerning the oracles for the token generation, we allow the adversary to adaptively query corrupted tokens via $\textsf{CorTokGen}$ and honest tokens via $\textsf{HonTokGen}$. The former mirrors attacks where the adversary gets complete control over tokens while the latter allows the adversary to query the generation of an honest token without access to the token itself.

Moreover, we also provide $$\textsf{Enc} '$$ and $$\textsf{HonUpdate} $$ oracles. Thereby, $$\textsf{Enc} '$$ allows generating honest ciphertexts (under $$mpk $$) and $$\textsf{HonUpdate} $$ allows updating ciphertexts which have been honestly^8^ generated via $$\textsf{Enc} '$$ without revealing the update token to the adversary. See that via $$\textsf{HonTokGen} $$, the adversary can query an honest token generation and the experiment can use such a token for the honest update.

The validity of the adversary is checked in the end of the security game. Essentially, the adversary is valid if and only if:

- the adversary cannot trivially distinguish the challenge ciphertext,
- the adversary has not received update tokens towards $$t $$ for the challenge ciphertexts where it has queried function keys under $$t $$ with $$f(x^*_0)\ne f(x^*_1)$$,
- the adversary has only queried updated challenge ciphertexts for which it has function keys that satisfy $f(x^*_0)=f(x^*_1)$.

If the adversary is valid and it has correctly guessed which message was encrypted in the challenge ciphertext, the adversary wins the game.

$\mathsf {IND\text {-}CUFE\text {-}CPA}$ *security* We say that a CUFE scheme is $\mathsf {IND\text {-}CUFE\text {-}CPA}$-secure if any PPT adversary succeeds in the following experiment only with probability negligibly larger than $1/2$. The experiment starts by computing the initial main public and secret key pair $(mpk,msk)\leftarrow \textsf{Setup} (\lambda ,\mathcal {F})$, initializes empty sets $\mathcal {K}$, $\mathcal {C}$, $\mathcal{U}\mathcal{C}$, $\mathcal{H}\mathcal{T}$, $\mathcal{C}\mathcal{T}$ to track keys, ciphertexts, updated ciphertexts, honest and corrupted tokens, respectively, as well as initializes the counters $\textsf{c}$, $\textsf{uc}$, $\textsf{ht}$, $\textsf{ct}$ for ciphertexts, updated ciphertexts, honest tokens and corrupted tokens, respectively.

**Fig. 1:**
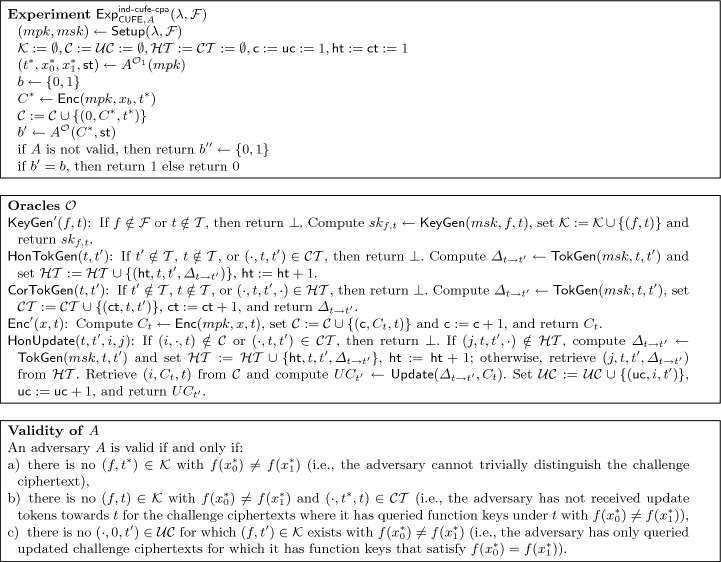
The $$\mathsf {IND\text {-}CUFE\text {-}CPA}$$ security notion for $$\textsf{CUFE} $$. If $$\mathcal {O}_1=\bot $$, then we call the experiment semi-adaptive, and if $$\mathcal {O}_1=\mathcal {O}$$, then we call it adaptive. If $$\mathcal {O}_1 = \bot $$ and $$mpk $$ is not initially given to $$A$$, then we call the experiment selective

At some point, the adversary outputs target tag and messages $(t ^*,x_0^*,x_1^*)$. Next, the experiment tosses a coin *b*, computes $C ^*\leftarrow \textsf{Enc} (mpk,x_b^*,t ^*)$, adds $(0,C ^*,t ^*)$ to $\mathcal {C}$, and gives $C ^*$ to the adversary. The adversary eventually outputs a guess $b'$, where the experiment returns 1 if $b'=b$ and the adversary is valid. In the adaptive security game the adversary has full access to all oracles from the beginning, whereas in the selective security game the adversary only gets access to the oracles after committing to the target tag $t ^*$ and challenge messages $(x_0^*,x_1^*)$. Figure 1 depicts the experiment.

### Definition 6

($\mathsf {IND\text {-}CUFE\text {-}CPA}$ security) A CUFE scheme $\textsf{CUFE}$ is $\mathsf {IND\text {-}CUFE\text {-}CPA}$-secure iff for any valid PPT adversary $A$ the advantage function

$$\begin{aligned} \textsf{Adv}^{\mathsf {ind\text {-}cufe\text {-}cpa}}_{\textsf{CUFE},A}(\lambda ,\mathcal {F}):=\left| \Pr \left[ {\textsf{Exp}^{\mathsf {ind\text {-}cufe\text {-}cpa}}_{\textsf{CUFE},A}(\lambda ,\mathcal {F})=1}\right] -1/2\,\right| , \end{aligned}$$

is negligible in $\lambda$, where $\textsf{Exp}^{\mathsf {ind\text {-}cufe\text {-}cpa}}_{\textsf{CUFE},A}$ is defined in Fig. 1.

### Remark 2

We model the experiment semi-adaptive (i.e., the target tag and messages are chosen by the adversary before it has access to oracles, but after it has seen the main public key) as well as adaptive (i.e., the adversary has access to the oracles before specifying target tag and messages). Note that in Fig. 1, we cover this by setting $\mathcal {O}_1=\bot$, i.e., the adversary has no access to oracles in the first phase, or $\mathcal {O}_1=\mathcal {O}$, i.e., the adversary has access to the oracles throughout both phases, respectively. We note that it would also be possible to define the experiment in a weaker selective setting or either choosing only the tag or the messages in a semi-adaptive sense. This is straightforward to model and we omit it for the sake of simplicity. Moreover, we note that to move from a selective to an adaptive setting, one can utilize the standard technique of complexity leveraging if one is willing to accept that message and/or tag spaces are polynomially bounded in the security parameter.

## Generic Construction of CUFE and Extensions

In this section, we present a generic construction of CUFE for any function from indistinguishability obfuscation that provides semi-adaptive $\mathsf {IND\text {-}CUFE\text {-}CPA}$ security. For the sake of consistency, we opt to present it for the equality predicate on tags and then extend the expressiveness of predicates beyond the equality testing on tags. We show that due to the way our construction is built, it easily supports any predicate that can be represented as a circuit of arbitrary polynomial size. Moreover, we conjecture that one can obtain adaptive FE security either using the black-box transformation of Ananth et al. [7] along with applying complexity leveraging over the tag space or by directly extending the adaptively secure FE construction of Waters [57] to the CUFE setting.

### Puncturable Tag-Based Deterministic Encryption

Our generic construction relies on a primitive called puncturable tag-based deterministic encryption (PTDE), which can be seen as a tag-based variant of puncturable deterministic encryption (PDE) introduced by Waters [57].

#### Definition 7

(Puncturable Tag-Based Deterministic Encryption) A puncturable tag-based deterministic encryption (PTDE) scheme $\Sigma$ with message space $\mathcal {M}$ and tag space $\mathcal {T}$ consists of the following algorithms: (possibly) randomized algorithms $\textsf{Setup}$ and $\textsf{Puncture}$, along with deterministic algorithms $\textsf{Enc}$ and $\textsf{Dec}$.

- $\textsf{Setup} (1^\lambda )$, on input a security parameter $1^\lambda$, outputs a key *k*.
- $\textsf{Enc} (k,t,m)$, on input a key *k*, a tag $t \in \mathcal {T}$ and a message *m*, outputs a ciphertext *c*.
- $\textsf{Dec} (k,t,c)$, on input a key *k*, a tag $t \in \mathcal {T}$ and a ciphertext *c*, outputs a message $m \in \mathcal {M} \cup \{\bot \}$.
- $\textsf{Puncture}(k,t,m_0,m_1)$, on input a key *k*, a tag $t \in \mathcal {T}$ and a pair of messages $m_0,m_1 \in \mathcal {M}$, outputs a new key $k^{t,m_0,m_1}$ (the superscript is used to indicate the tag and messages where the key is punctured).

*Correctness* We say that a PTDE scheme $\Sigma$ is correct if there exists a negligible function $\textsf{negl}$, such that for all $\lambda \in \mathbb {N}$, for all $t \in \mathcal {T}$, for all pairs of messages $m_0,m_1 \in \mathcal {M}$, for all $k \leftarrow \Sigma .\textsf{Setup} (1^\lambda )$ and $k^{t,m_0,m_1} \leftarrow \Sigma .\textsf{Puncture}(k,t,m_0,m_1)$, for all $m \ne m_0,m_1$, it holds that

$$\begin{aligned} \Pr \left[ {\Sigma .\textsf{Dec} (k^{t,m_0,m_1},\Sigma .\textsf{Enc} (k,t,m)) \ne m}\right] = \textsf{negl}(\lambda ). \end{aligned}$$

Moreover, we have that for all *m* (including $m_0,m_1$),

$$\begin{aligned} \Pr \left[ {\Sigma .\textsf{Dec} (k,\Sigma .\textsf{Enc} (k,t,m)) \ne m}\right] = \textsf{negl}(\lambda ). \end{aligned}$$

Next, we recall the notion of (selective) indistinguishability security for PTDE.

#### Definition 8

(Indistinguishability Security for PTDE) A PTDE scheme $$\Sigma $$ is indistinguishability secure, if for all PPT adversaries $$A $$ it holds that

$$\begin{aligned} \textsf{Adv}_{\Sigma ,A}(\lambda ):= \left| \Pr \left[ { \begin{array}{l} k \leftarrow \Sigma .\textsf{Setup} (1^\lambda ), \\ (t, m_0,m_1) \leftarrow A(1^\lambda ), \\ k^{t,m_0,m_1} \leftarrow \Sigma .\textsf{Puncture}(k,t,m_0,m_1) \\ b \leftarrow \{0,1\} \\ c_0 \leftarrow \Sigma .\textsf{Enc} (k,t,m_b), c_1 \leftarrow \Sigma .\textsf{Enc} (k,t,m_{1-b}) \\ b^* \leftarrow A(k^{t,m_0,m_1},c_0,c_1) :\\ b = b^* \end{array} }\right] - \frac{1}{2} \right| , \end{aligned}$$

is negligible.

#### Remark 3

Our definition allows for a key to be punctured at two messages and a tag, which extends the original PDE definition given in [57] with a tag puncturing. We note that this differs from puncturable tag-based encryption given by Chvojka et al. [23], which allows puncturing only at tags instead and constitutes a randomized encryption scheme.

#### Construction of PTDE

We extend the PDE construction given by Waters [57] to additionally consider tags. Our PTDE scheme has message space $\mathcal {M} = \{0,1\}^\lambda$ and tag space $\mathcal {T} = \{0,1\}^\ell$. We make use of two (puncturable) PRF families, where the first one is an injective puncturable PRF $F_1$ that takes inputs from $\lambda$ bits to $\ell = \ell (\lambda )$ bits, and the second one $F_2$ takes inputs from $\ell$ bits to $\lambda$ bits. The construction is as follows.

- $\textsf{Setup} (1^\lambda )$: Sample keys $k_1 \leftarrow \textsf{PRF}.\textsf{Gen} _{F_1}(1^\lambda )$ and $k_2 \leftarrow \textsf{PRF}.\textsf{Gen} _{F_2}(1^\lambda )$, and output $k:= (k_1, k_2)$.
- $\textsf{Enc} (k:= (k_1, k_2), t, m)$: Deterministically compute and output a ciphertext
  $$\begin{aligned} c := (c_1 = F_1(k_1, m), c_2 = F_2(k_2, c_1 \oplus t) \oplus m). \end{aligned}$$
- $\textsf{Dec} (k:= (k_1, k_2), t, c:= (c_1, c_2))$: Compute $m' = F_2(k_2, c_1 \oplus t) \oplus c_2$. If $F_1(k_1, m') = c_1$, then output $m'$, otherwise output $\bot$.
- $\textsf{Puncture}(k:= (k_1, k_2), t, m_0, m_1)$: Compute $d = F_1(k_1,m_0)$ and $e = F_1(k_1,m_1)$. Compute $k_{1}^{m_0,m_1} \leftarrow \textsf{PRF}.\textsf{Puncture}_{F_1}(k_1, \{m_0,m_1\})$ and $k_{2}^{t} \leftarrow \textsf{PRF}.\textsf{Puncture}_{F_2}(k_2,\{d \oplus t,e \oplus t\})$, and output $k^{t,m_0,m_1}:= (k_{1}^{m_0,m_1},k_{2}^{t})$.

The correctness for the non-punctured keys follows by observation, and correctness for key $k^{t,m_0,m_1}$ on all messages $m \ne m_0,m_1$ holds as long as $F_1(k_1,m) \ne F_1(k_1,m_0)$ or $F_1(k_1,m_1)$, which holds because $F_1$ is injective. The security follows straightforwardly from the (punctured) PRF security of $F_1$ and $F_2$ and is established with the following theorem.

##### Theorem 1

Let $F_1$ and $F_2$ be secure puncturable pseudorandom functions. Then, our construction is (selectively) indistinguishable secure PTDE scheme.

##### Proof

The security proof follows via a sequence of hybrid games. Hereafter, let $\textsf{Game} _{i} \approx \textsf{Game} _{i+1}$ denote $\left| \Pr [\textsf{Game} _{i} = 1] - \Pr [\textsf{Game} _{i+1} = 1]\right| \le \textsf{negl}(\lambda )$.

- $\textsf{Game} _{0}:$ This corresponds to the honest execution of the (selective) indistinguishability game of PTDE.
- $\textsf{Game} _{1}:$ This is identical to $\textsf{Game} _{0}$ with the exception that the challenger randomly chooses $c_1^b, c_1^{1-b}$ (when computing the challenge ciphertext) instead of computing $c_1^b = F_1(k_1, m_b)$ and $c_1^{1-b} = F_1(k_1, m_{1-b})$.
- $\textsf{Game} _{2}:$ This is identical to $\textsf{Game} _{1}$ with the exception that the challenger randomly chooses $c_2^b, c_2^{1-b}$ (when computing the challenge ciphertext) instead of computing $c_2^b = F_2(k_2, c_1^b \oplus t)$ and $c_2^{1-b} = F_2(k_2, c_2^{1-b} \oplus t)$.

##### Lemma 1

If $F_1$ is a selectively secure puncturable PRF, then it holds that $\textsf{Game} _{0} \approx \textsf{Game} _{1}$.

##### Proof

We describe a PPT reduction algorithm $$B $$ that plays the selective puncturable tag-based deterministic encryption (PTDE) security game. $$B $$ receives $$(t,m_0,m_1)$$ from $$A $$ and proceeds as in $$\textsf{Game} _{0}$$, except that it samples a bit $$b \in \{0,1\}$$, submits $$m_b,m_{1-b}$$ to the punctured PRF challenger. $$B $$ receives back a punctured PRF key $$k_{\textsf{prf}}^{m_b,m_{1-b}}$$ and challenge values $$z_0,z_1$$. $$B $$ sets $$k_{\textsf{ptde}}:= (k_{\textsf{prf}}^{m_b,m_{1-b}}, k_{\textsf{prf},2})$$, $$c_0:= (z_b, F_2(k_{\textsf{prf},2}, z_b \oplus t) \oplus m_b)$$ and $$c_1:= (z_{1-b}, F_2(k_{\textsf{prf},2}, z_{1-b} \oplus t) \oplus m_{1-b})$$ and returns $$(k_{\textsf{ptde}}, c_0, c_1)$$ to $$A $$. If $$A $$ wins, i.e., $$b' = b$$, then $$B $$ outputs 1 to indicate that $$z_0 = F_1(k_{\textsf{prf},1},m_0)$$ and $$z_1 = F_1(k_{\textsf{prf},1},m_1)$$, for some PRF key $$k_{\textsf{prf},1}$$, and otherwise, it outputs 0 to indicate that $$z_0, z_1$$ were random values.

We observe that if $$z_0,z_1$$ are generated as $$z_0 = F_1(k_{\textsf{prf},1},m_0)$$ and $$z_1 = F_1(k_{\textsf{prf},1},m_1)$$, then $$B $$ gives the view of $$\textsf{Game} _{0}$$ to $$A $$. Otherwise, if $$z_0$$ and $$z_1$$ were chosen randomly, then the view is of $$\textsf{Game} _{1}$$. Therefore, if $$A $$ can distinguish between the two games with non-negligible advantage, then $$B $$ must also have non-negligible advantage against the puncturable PRF security game. $$\square $$

##### Lemma 2

If $F_2$ is a selectively secure puncturable PRF, then it holds that $\textsf{Game} _{1} \approx \textsf{Game} _{2}$.

##### Proof

The proof of this lemma follows analogously to that of Lemma 1. $\square$

We note that since $c_1^b, c_1^{1-b}, c_2^b, c_2^{1-b}$ are all chosen uniformly at random in $\textsf{Game} _{2}$, we have that the challenge ciphertexts $c_b:= (c_1^b, c_2^b)$, for $b \in \{0,1\}$, information-theoretically hide the bit *b*. This final information-theoretic argument depends on the fact that the distribution of $\textsf{PRF}.\textsf{Puncture}_{F_1}(k_{\textsf{prf},1}, \{m_0,m_1\})$ is the same as $\textsf{PRF}.\textsf{Puncture}_{F_1}(k_{\textsf{prf},1}, \{m_1,m_0\})$. This concludes the proof of Theorem 1.$\square$

### Generic CUFE from iO for any Function

The generic construction is inspired by the punctured programming approach to construct functional encryption from indistinguishability obfuscation, given by Waters [57]. More precisely, the construction makes use of indistinguishability obfuscation $i\mathcal {O}$, puncturable tag-based deterministic encryption (PTDE) scheme $\Sigma$, puncturable pseudorandom function *F* and pseudorandom generator $\textsf{PRG}$. The construction is described below (where the parts in blue in programs PInit:2, PKey:2 and PUpdate:2 highlight the changes with respect to programs PInit:1, PKey:1 and PUpdate:1):

- $\textsf{Setup} (1^\lambda , \mathcal {F})$: Compute the following:
  1. Sample $$k_{\textsf{prf},o} \leftarrow \textsf{PRF}.\textsf{Gen} _F(1^\lambda )$$ and $$k_{\textsf{prf},u} \leftarrow \textsf{PRF}.\textsf{Gen} _F(1^\lambda )$$.
  2. Compute an obfuscation $P_{pp} \leftarrow i\mathcal {O}(\text {PInit:1}[k_{\textsf{prf},o}])$ for the program $\text {PInit:1}[k_{\textsf{prf},o}]$^9^.
  Output the main public/secret key pair $(mpk:= P_{pp}$, $msk:= (k_{\textsf{prf},o},k_{\textsf{prf},u}))$.
- $\textsf{KeyGen} (msk:= (k_{\textsf{prf},o},k_{\textsf{prf},u}), f, t)$: Compute an obfuscation $P_{f,t} \leftarrow i\mathcal {O}(\text {PKey:1}[k_{\textsf{prf},o},k_{\textsf{prf},u},f,t])$ for the program $\text {PKey:1}[k_{\textsf{prf},o},k_{\textsf{prf},u},f,t]$.^10^ Output the secret key $sk_{f,t}:= P_{f,t}$.
- $\textsf{TokGen} (msk:= (k_{\textsf{prf},o},k_{\textsf{prf} _u}),t,t')$: Compute an obfuscation $P_{t \rightarrow t'} \leftarrow i\mathcal {O}(\text {PUpdate:1}[k_{\textsf{prf},o},k_{\textsf{prf},u},t,t'])$ for the program $\text {PUpdate:1}[k_{\textsf{prf},o},k_{\textsf{prf},u},t,t']$^11^. Output the update token $\Delta _{t \rightarrow t'}:= P_{t \rightarrow t'}$.
- $\textsf{Enc} (mpk:= P_{pp},m,t)$: Compute the following:
  1. Sample a random $$r \in \{0,1\}^\lambda $$.
  2. Run the obfuscated program $(p, k_{\textsf{ptde}}) \leftarrow P_{pp}(r)$.
  3. Compute $$c \leftarrow \Sigma .\textsf{Enc} (k_{\textsf{ptde}},t,m)$$.
  Output the ciphertext $C_t:= (p, c)$.
- $\textsf{Update} (\Delta _{t \rightarrow t'}:= P_{t \rightarrow t'}, C_t)$: Compute the following:
  1. Sample a random $$r \in \{0,1\}^\lambda $$.
  2. Run the obfuscated program $C_{t'} \leftarrow P_{t \rightarrow t'}(C_t, r)$.
  Output the updated ciphertext $C_{t'}$.^12^
- $\textsf{Dec} (sk _{f,t}:= P_{f,t}, C_t)$: Run the obfuscated program $f(m) \leftarrow P_{f,t}(C_t)$ and output *f*(*m*).

*Correctness* The correctness of our construction follows straightforwardly from the correctness of the puncturable tag-based deterministic encryption scheme $\Sigma$, puncturable pseudorandom function *F*, pseudorandom generator $\textsf{PRG}$, obfuscator $i\mathcal {O}$, and the description of the programs PInit:1, PKey:1 and PUpdate:1.
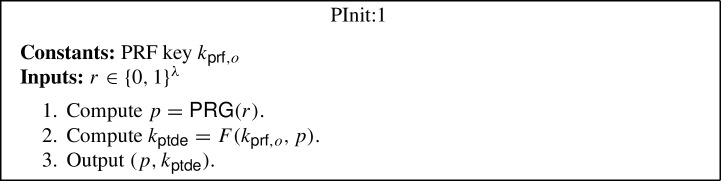

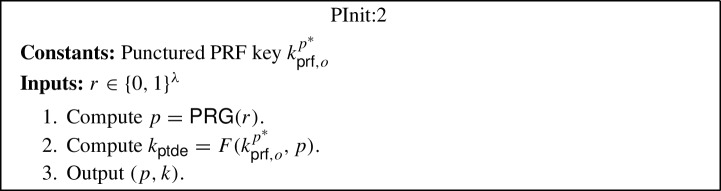

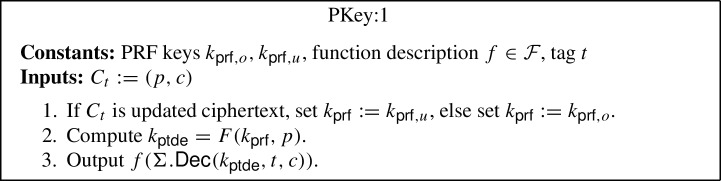

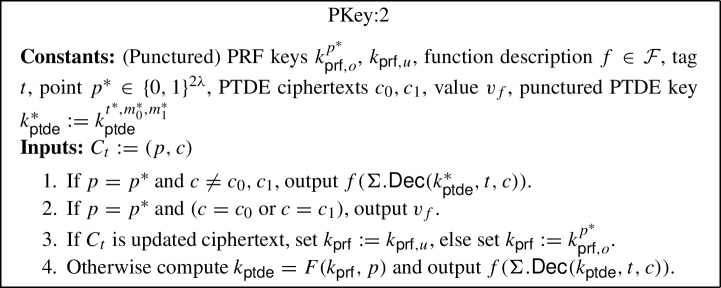

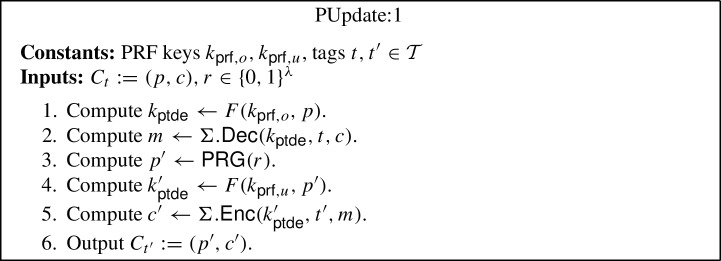

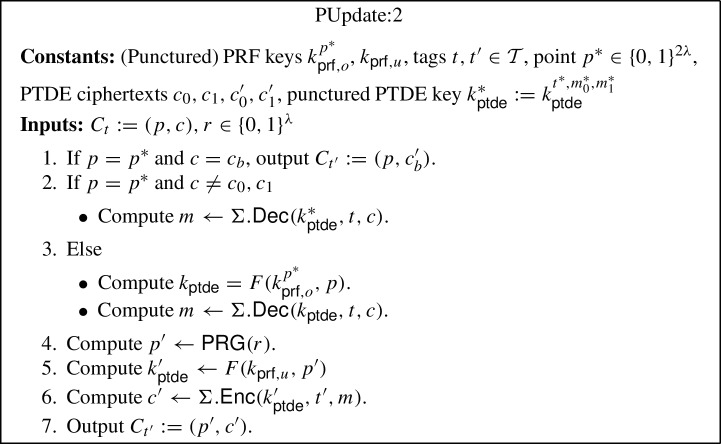


Next, we present the proof of $\mathsf {IND\text {-}CUFE\text {-}CPA}$ security of our generic construction.

#### Theorem 2

Let $\Sigma$ be a puncturable tag-based deterministic encryption scheme, *F* be a secure puncturable pseudorandom function, $\textsf{PRG}$ be a secure pseudorandom generator, $i\mathcal {O}$ be an indistinguishability obfuscator for the circuit class $\mathcal {C} _\lambda$. Then, our generic construction is a semi-adaptively $\mathsf {IND\text {-}CUFE\text {-}CPA}$-secure CUFE scheme.

#### Proof

The proof is organized in a sequence of hybrid games, where initially the challenger encrypts $m_b$ for a random bit $b \in \{0,1\}$, and we gradually (in multiple steps) change the encryption into an encryption of $m_0$, which is independent of the bit *b*. We first define the sequence of games, and then, show (based on the security of different primitives) that any PPT adversary’s advantage in each game must be negligibly close to the previous game. Hereafter, let $\textsf{Game} _{i} \approx \textsf{Game} _{i+1}$ denote $\left| \Pr [\textsf{Game} _{i} = 1] - \Pr [\textsf{Game} _{i+1} = 1]\right| \le \textsf{negl}(\lambda )$.

- $$\textsf{Game} _{0}$$: This corresponds to the honest execution of the semi-adaptive variant of the indistinguishability game given in Sect. 3. More precisely, the adversary is given the main public key $$mpk $$, then the adversary selects a challenge tag $$t^*$$ and a challenge message pair $$m_0^*, m_1^*$$, and the challenger choses a bit $$b \in \{0,1\}$$ and encrypts $$m_b^*$$ in the challenge ciphertext.
- $\textsf{Game} _{1}$: This is identical to $\textsf{Game} _0$ with the exception that the challenger choses a random $p^* \in \{0,1\}^{2\lambda }$ during the computation of the challenge ciphertext, instead of choosing a random $r^* \in \{0,1\}^\lambda$ and computing $p^* \leftarrow \textsf{PRG} (r^*)$.
- $\textsf{Game} _2$: This is identical to $\textsf{Game} _1$ with the exception that the challenger computes the punctured key $k^{p^*}_{\textsf{prf},o} \leftarrow \textsf{PRF}.\textsf{Puncture}_{F}(k_{\textsf{prf},o},p^*)$ and sets $P_{pp} \leftarrow i\mathcal {O}(\text {PInit:2}[k_{\textsf{prf},o}^{p^*}])$.
- $\textsf{Game} _3$: This is identical to $\textsf{Game} _2$ with the exception that for answering each secret key query $(f, t) \in (\mathcal {F} \times \mathcal {T})$, the challenger does the following: Compute $k_{\textsf{ptde}}' \leftarrow \Sigma .\textsf{Puncture}(k_{\textsf{ptde}}^*,t^*,m_0^*,m_1^*)$, for $k_{\textsf{ptde}}^* \leftarrow F(k_{\textsf{prf},o},p^*)$, compute $c_0' \leftarrow \Sigma .\textsf{Enc} (k_{\textsf{ptde}}^*, t, m_0^*)$, $c_1' \leftarrow \Sigma .\textsf{Enc} (k_{\textsf{ptde}}^*, t, m_1^*)$, and $v_f = f(m_0^*) = f(m_1^*)$. Then, let $c_0,c_1$ consist of $c_0',c_1'$ in lexicographic order,^13^ and the challenger responds with $P_{f,t} \leftarrow i\mathcal {O}(\text {PKey:2}[k_{\textsf{prf},o}^{p^*},k_{\textsf{prf},u},f,t,p^*,c_0,c_1,v_f,k_{\textsf{ptde}}'])$.
- $\textsf{Game} _4$: This is identical to $\textsf{Game} _3$ with the exception that for answering each token generation query $(t, t') \in (\mathcal {T} \times \mathcal {T})$, the challenger does the following: Compute $k_{\textsf{ptde}}' \leftarrow \Sigma .\textsf{Puncture}(k_{\textsf{ptde}}^*,t^*,m_0^*,m_1^*)$, for $k_{\textsf{ptde}}^* \leftarrow F(k_{\textsf{prf},o},p^*)$, compute $c_0 \leftarrow \Sigma .\textsf{Enc} (k_{\textsf{ptde}}^*, t, m_0^*)$, $c_1 \leftarrow \Sigma .\textsf{Enc} (k_{\textsf{ptde}}^*, t, m_1^*)$, and $c_0' \leftarrow \Sigma .\textsf{Enc} (k_{\textsf{ptde}}'', t', m_0^*)$, $c_1' \leftarrow \Sigma .\textsf{Enc} (k_{\textsf{ptde}}'', t', m_1^*)$, for $k_{\textsf{ptde}}'' \leftarrow F(k_{\textsf{prf},u},r)$ and random $r \in \{0,1\}^{\lambda }$. Then, sort and order $c_0,c_1,c_0',c_1'$ lexicographically,^14^ and respond with $P_{t \rightarrow t'} \leftarrow i\mathcal {O}(\text {PUpdate:2}[k_{\textsf{prf},o}^{p^*},k_{\textsf{prf},u},,t,t',p^*,c_0,c_1,c_0',c_1',k_{\textsf{ptde}}'])$.
- $\textsf{Game} _5$: This is identical to $\textsf{Game} _4$ with the exception that the challenger samples a random $k_{\textsf{ptde}}^*$ instead of computing it as $k_{\textsf{ptde}}^* \leftarrow F(k_{\textsf{prf},o},p^*)$.
- $\textsf{Game} _6$: This is identical to $\textsf{Game} _5$ with the exception that the challenger encrypts $m_0^*$, i.e., the challenger computes $c^* \leftarrow \Sigma .\textsf{Enc} (k_{\textsf{ptde}}^*,t^*,m_0^*)$ and outputs $(p^*, c^*)$.

$$\square $$

#### Lemma 3

If $\textsf{PRG}$ is a secure pseudorandom generator, then it holds that $\textsf{Game} _{0} \approx \textsf{Game} _{1}$.

#### Proof

We describe a PPT reduction algorithm $$B $$ that plays the PRG security game. First, $$B $$ creates the main public/secret key pair $$(mpk,msk)$$ (as in $$\textsf{Game} _{0}$$). Next, $$B $$ receives a PRG challenge $$p \in \{0,1\}^{2\lambda }$$. Then, $$B $$ runs the adversary $$A $$ and executes the CUFE security game (as described in $$\textsf{Game} _{0}$$), with the exception that when computing the challenge ciphertext it sets $$p^*:= p$$. We note that since $$B $$ generates everything else itself (as in $$\textsf{Game} _{0}$$, it has all the necessary information to answer the oracle queries of $$A $$). Lastly, if $$A $$ wins, i.e., $$b' = b$$, then $$B $$ outputs 1 to indicate that *p* was in the image of $$\textsf{PRG} $$, and otherwise, it outputs 0 to indicate that *p* was chosen randomly.

We observe that if the PRG challenger generated $$p = \textsf{PRG} (r)$$, for some $$r \in \{0,1\}^\lambda $$, then $$B $$ gives the view of $$\textsf{Game} _{0}$$ to $$A $$. Otherwise, if *p* was chosen randomly, then the view is of $$\textsf{Game} _{1}$$. Therefore, if $$A $$ can distinguish between the two games with non-negligible advantage, then $$B $$ must also have non-negligible advantage against the PRG security game. $$\square $$

#### Lemma 4

If $i\mathcal {O}$ is an indistinguishability obfuscator for the circuit class $\mathcal {C} _\lambda$, then it holds that $\textsf{Game} _{1} \approx \textsf{Game} _{2}$.

#### Proof

We construct a distinguisher $$B $$ for $$i\mathcal {O}$$. $$B $$ proceeds as in $$\textsf{Game} _{1}$$, with the exception that it computes the punctured PRF key $$k^{p^*}_{\textsf{prf},o} \leftarrow \textsf{PRF}.\textsf{Puncture}_{F}(k_{\textsf{prf},o},p^*)$$ and generates the two circuits $$C_0 = \text {PInit:1}[k_{\textsf{prf},o}]$$ and $$C_1 = \text {PInit:2}[k_{\textsf{prf},o}^{p^*}]$$. $$B $$ submits $$C_0,C_1$$ to the $$i\mathcal {O}$$ challenger and receives back a program *P*, which it sets as $$mpk:= P_{pp}:= P$$, and returns it to the CUFE adversary $$A $$. The rest of the execution is identical to $$\textsf{Game} _{1}$$. If $$A $$ wins, i.e., $$b' = b$$, then $$B $$ outputs 0 to indicate that *P* was an obfuscation of $$C_0$$, and otherwise, it outputs 1 to indicate that *P* was an obfuscation of $$C_1$$.

We observe that if the $$i\mathcal {O}$$ challenger generated *P* as an obfuscation of $$C_0$$, then $$B $$ gives the view of $$\textsf{Game} _{1}$$ to $$A $$. Otherwise, if *P* was generated as an obfuscation of $$C_1$$, then the view is that of $$\textsf{Game} _{2}$$. Moreover, the programs are functionally equivalent with all but negligible probability, because $$p^*$$ lies outside the image of $$\textsf{PRG} $$ with probability at least $$1 - 2^\lambda $$. Therefore, if $$A $$ can distinguish between the two games with non-negligible advantage, then $$B $$ must also have non-negligible advantage against the $$i\mathcal {O}$$ security game for the circuit class $$\mathcal {C} _\lambda $$. $$\square $$

#### Lemma 5

If $i\mathcal {O}$ is an indistinguishability obfuscator for the circuit class $\mathcal {C} _\lambda$, then it holds that $\textsf{Game} _{2} \approx \textsf{Game} _{3}$.

#### Proof

To prove this lemma, we consider a hybrid argument. Let $$Q_k = Q_k(\lambda )$$ denote the number of secret key queries issued by the CUFE adversary $$A $$. For $$i \in [0,Q_k]$$, we define $$\textsf{Game} _{2,i}$$ to be equivalent to $$\textsf{Game} _{2}$$ with the exception that the first *i* secret key queries are handled as in $$\textsf{Game} _{3}$$ and the last $$Q_k - i$$ are handled as in $$\textsf{Game} _{2}$$. Note that $$\textsf{Game} _{2,0}$$ is the same as $$\textsf{Game} _{2}$$ and $$\textsf{Game} _{2,Q_k}$$ is the same as $$\textsf{Game} _{3}$$. Hence, to prove security we need to establish that no adversary can distinguish between $$\textsf{Game} _{2,i}$$ and $$\textsf{Game} _{2,i+1}$$, for $$i \in [0,Q_k-1]$$, with non-negligible advantage.

We construct a distinguisher $$B $$ for $$i\mathcal {O}$$. $$B $$ proceeds as in $$\textsf{Game} _{2}$$, except that the first *i* secret key queries are answered as in $$\textsf{Game} {3}$$. For query $$i + 1$$, $$B $$ computes $$k_{\textsf{ptde}}' \leftarrow \Sigma .\textsf{Puncture}(k_{\textsf{ptde}}^*,t^*,m_0^*,m_1^*)$$, for $$k_{\textsf{ptde}}^* \leftarrow F(k_{\textsf{prf},o},p^*)$$, computes $$c_0' \leftarrow \Sigma .\textsf{Enc} (k_{\textsf{ptde}}^*, t, m_0^*)$$, $$c_1' \leftarrow \Sigma .\textsf{Enc} (k_{\textsf{ptde}}^*, t, m_1^*)$$, and $$v_f = f(m_0^*) = f(m_1^*)$$, where *f* and *t* are the queried function and tag, respectively. Then, let $$c_0,c_1$$ consist of $$c_0',c_1'$$ in lexicographic order, $$B $$ generates the two circuits $$C_0 = \text {PKey:1}[k_{\textsf{prf},o},k_{\textsf{prf},u},f,t]$$ and $$C_1 = \text {PKey:2}[k_{\textsf{prf},o}^{p^*},k_{\textsf{prf},u},f,t,p^*,c_0,c_1,v_f,k_{\textsf{ptde}}']$$. $$B $$ submits $$C_0,C_1$$ to the $$i\mathcal {O}$$ challenger and receives back a program *P*, which it sets as $$sk _{f,t}:= P_{f,t}:= P$$, and returns it to the CUFE adversary $$A $$ as the query answer. If $$A $$ wins, i.e., $$b' = b$$, then $$B $$ outputs 0 to indicate that *P* was an obfuscation of $$C_0$$, and otherwise, it outputs 1 to indicate that *P* was an obfuscation of $$C_1$$.

We observe that if the $$i\mathcal {O}$$ challenger generated *P* as an obfuscation of $$C_0$$, then $$B $$ gives the view of $$\textsf{Game} _{2,i}$$ to $$A $$. Otherwise, if *P* was generated as an obfuscation of $$C_1$$, then the view is that of $$\textsf{Game} _{2,i+1}$$. Moreover, the programs are functionally equivalent with all but negligible probability, because the only difference in the programs is that the response is hardwired for the two inputs (i.e., for the challenge ciphertexts). Therefore, if $$A $$ can distinguish between the two games with non-negligible advantage, then $$B $$ must also have non-negligible advantage against the $$i\mathcal {O}$$ security game for the circuit class $$\mathcal {C} _\lambda $$. $$\square $$

#### Lemma 6

If $i\mathcal {O}$ is an indistinguishability obfuscator for the circuit class $\mathcal {C} _\lambda$, then it holds that $\textsf{Game} _{3} \approx \textsf{Game} _{4}$.

#### Proof

To prove this lemma, we consider a hybrid argument. Let $$Q_t$$ denote the total number of token generation queries issued by the CUFE adversary $$A $$, where $$Q_t = Q_{ht} + Q_{ct}$$, such that $$Q_{ht} = Q_{ht}(\lambda )$$ and $$Q_{ct} = Q_{ct}(\lambda )$$ denote the number of honest and corrupted token generation queries, respectively. For $$i \in [0,Q_t]$$, we define $$\textsf{Game} _{3,i}$$ to be equivalent to $$\textsf{Game} _{3}$$ with the exception that the first *i* token generation queries are handled as in $$\textsf{Game} _{4}$$ and the last $$Q_t - i$$ are handled as in $$\textsf{Game} _{3}$$. Note that $$\textsf{Game} _{3,0}$$ is the same as $$\textsf{Game} _{3}$$ and $$\textsf{Game} _{3,Q_t}$$ is the same as $$\textsf{Game} _{4}$$. Hence, to prove security we need to establish that no adversary can distinguish between $$\textsf{Game} _{3,i}$$ and $$\textsf{Game} _{3,i+1}$$, for $$i \in [0,Q_t-1]$$, with non-negligible advantage.

We construct a distinguisher $$B $$ for $$i\mathcal {O}$$. $$B $$ proceeds as in $$\textsf{Game} _{3}$$, except that the first *i* token generation queries are answered as in $$\textsf{Game} _{4}$$. For query $$i + 1$$, $$B $$ computes $$k_{\textsf{ptde}}' \leftarrow \Sigma .\textsf{Puncture}(k_{\textsf{ptde}}^*,t^*,m_0^*,m_1^*)$$, for $$k_{\textsf{ptde}}^* \leftarrow F(k_{\textsf{prf},o},p^*)$$, computes $$c_0 \leftarrow \Sigma .\textsf{Enc} (k_{\textsf{ptde}}^*, t, m_0^*)$$, $$c_1 \leftarrow \Sigma .\textsf{Enc} (k_{\textsf{ptde}}^*, t, m_1^*)$$, and $$c_0' \leftarrow \Sigma .\textsf{Enc} (k_{\textsf{ptde}}'', t', m_0^*)$$, $$c_1' \leftarrow \Sigma .\textsf{Enc} (k_{\textsf{ptde}}'', t', m_1^*)$$, for $$k_{\textsf{ptde}}'' \leftarrow F(k_{\textsf{prf},u},r)$$ and random $$r \in \{0,1\}^{\lambda }$$, where $$t,t'$$ are the queried tags. Then, $$B $$ sorts and orders $$c_0,c_1,c_0',c_1'$$ lexicographically. $$B $$ generates the two circuits $$C_0 = \text {PUpdate:1}[k_{\textsf{prf},o},k_{\textsf{prf},u},t,t']$$, and $$C_1 = \text {PUpdate:2}[k_{\textsf{prf},o}^{p^*},k_{\textsf{prf},u},t,t',p^*,c_0,c_1,c_0',c_1',k_{\textsf{ptde}}']$$. $$B $$ submits $$C_0,C_1$$ to the $$i\mathcal {O}$$ challenger and receives back a program *P*, which it sets as $$\Delta _{t \rightarrow t'}:= P_{t \rightarrow t'}:= P$$. If the query was a corrupted token generation query, then $$B $$ sends $$\Delta _{t \rightarrow t'}$$ to the CUFE adversary $$A $$ as the query answer, and otherwise, it stores it locally. If $$A $$ wins, i.e., $$b' = b$$, then $$B $$ outputs 0 to indicate that *P* was an obfuscation of $$C_0$$, and otherwise, it outputs 1 to indicate that *P* was an obfuscation of $$C_1$$.

We observe that if the $$i\mathcal {O}$$ challenger generated *P* as an obfuscation of $$C_0$$, then $$B $$ gives the view of $$\textsf{Game} _{3,i}$$ to $$A $$. Otherwise, if *P* was generated as an obfuscation of $$C_1$$, then the view is of $$\textsf{Game} _{3,i+1}$$. Moreover, the programs are functionally equivalent with all but negligible probability, because the only difference in the programs is that the response is hardwired for the two inputs (i.e., for the challenge ciphertexts). Therefore, if $$A $$ can distinguish between the two games with non-negligible advantage, then $$B $$ must also have non-negligible advantage against the $$i\mathcal {O}$$ security game for the circuit class $$\mathcal {C} _\lambda $$.$$\square $$

#### Lemma 7

If *F* is a selectively secure puncturable PRF, then it holds that $\textsf{Game} _{4} \approx \textsf{Game} _{5}$.

#### Proof

We describe a PPT reduction algorithm $$B $$ that plays the selective puncturable PRF security game. $$B $$ proceeds as in $$\textsf{Game} _{4}$$ in its interaction with the CUFE adversary $$A $$, except that it chooses a random $$p^* \in \{0,1\}^{2\lambda }$$ and submits it to the punctured PRF challenger. $$B $$ receives back a punctured PRF key $$k_{\textsf{prf}}^{p^*}$$ and a challenge value *z*. $$B $$ sets $$k_{\textsf{ptde}}^*:= z$$ and uses the punctured PRF key $$k_{\textsf{prf}}^{p^*}$$ to compute the challenge ciphertext and answer the oracle queries of $$A $$ as in $$\textsf{Game} _{4}$$. If $$A $$ wins, i.e., $$b' = b$$, then $$B $$ outputs 1 to indicate that $$z = F(k_{\textsf{prf}},p^*)$$, for some PRF key $$k_{\textsf{prf}}$$, and otherwise, it outputs 0 to indicate that *z* was a random value.

We observe that if *z* is generated as $$F(k_{\textsf{prf}},p^*)$$, then $$B $$ gives the view of $$\textsf{Game} _{4}$$ to $$A $$. Otherwise, if *z* was chosen randomly, then the view is that of $$\textsf{Game} _{5}$$. Therefore, if $$A $$ can distinguish between the two games with non-negligible advantage, then $$B $$ must also have non-negligible advantage against the puncturable PRF security game. $$\square $$

#### Lemma 8

If $\Sigma$ is a selectively secure puncturable tag-based deterministic encryption scheme, then it holds that $\textsf{Game} _{5} \approx \textsf{Game} _{6}$.

#### Proof

We note that the only difference between $$\textsf{Game} _{5}$$ and $$\textsf{Game} _{6}$$ is that in $$\textsf{Game} _{6}$$ the CUFE challenger always encrypts $$m_0^*$$, whereas in $$\textsf{Game} _{5}$$ the encrypted message could be $$m_0^*$$ or $$m_1^*$$, depending on the coin flip *b*. Moreover, when $$b = 0$$, the views of these two games are identical. Hence, if there is any difference in adversary $$A $$’s advantage in guessing *b* between $$\textsf{Game} _{5}$$ and $$\textsf{Game} _{6}$$ it must be solely conditioned on $$b = 1$$.

We describe a PPT reduction algorithm $$B $$ that plays the selective puncturable tag-based deterministic encryption (PTDE) security game. $$B $$ proceeds as in $$\textsf{Game} _{5}$$, except that it submits the challenge messages $$m_0^*, m_1^*$$ and tag $$t^*$$ (given by $$A $$) to the PTDE challenger, which replies with a punctured PTDE key $$k_{\textsf{ptde}}' \leftarrow \Sigma .\textsf{Puncture}(k_{\textsf{ptde}}^*,t^*,m_0^*,m_1^*)$$ and two ciphertexts $$c_0', c_1'$$. $$B $$ sets the challenge CUFE ciphertext to $$C_{t^*}:= (p^*, c^*:= c_0')$$.

Let $$c_0,c_1$$ consist of $$c_0',c_1'$$ in lexicographic order. Then, for answering each secret key query (of the form (*f*, *t*)), $$B $$ computes $$v_f = f(m_0^*) = f(m_1^*)$$, and uses the punctured PTDE key $$k_{\textsf{ptde}}'$$ to construct $$P_{f,t}:= \text {PKey:2}[k_{\textsf{prf},o}^{p^*},k_{\textsf{prf},u},f,t,p^*,c_0,c_1,v_f,k_{\textsf{ptde}}']$$. Similarly, for answering each token generation query (of the form $$(t,t')$$), $$B $$ guesses a $$\gamma \in \{0,1\}$$, computes $$c_{\gamma }'' \leftarrow \Sigma .\textsf{Enc} (k_{\textsf{ptde}}'', t', m_{\gamma }^*)$$, $$c_{1-\gamma }' \leftarrow \Sigma .\textsf{Enc} (k_{\textsf{ptde}}'', t', m_{1-\gamma }^*)$$, for $$k_{\textsf{ptde}}'' \leftarrow F(k_{\textsf{prf},u},r)$$ and random $$r \in \{0,1\}^{\lambda }$$. Then, $$B $$ uses the previously computed values and the punctured PTDE key $$k_{\textsf{ptde}}'$$ to construct $$P_{t \rightarrow t'}:= \text {PUpdate:2}[k_{\textsf{prf},o}^{p^*},k_{\textsf{prf},u},t,t',p^*,c_0,c_1,c_0'',c_1'',k_{\textsf{ptde}}']$$ and answer the token generation query. We note here that the guessed $$\gamma $$ incurs a 1/2 security loss. Encryption queries are answered in a straightforward way using the program $$\text {PInit:2}[k_{\textsf{prf},o}^{p^*}]$$.

Lastly, if $$A $$ wins, i.e., $$b' = b$$, then $$B $$ outputs 1 to indicate that $$c^*:= c_0'$$ was an encryption of $$m_1^*$$, and otherwise, it outputs 0 to indicate that $$c^*:= c_0'$$ was an encryption of $$m_0^*$$.

We observe that if $$c^*:= c_0'$$ is generated as $$\Sigma .\textsf{Enc} (k_{\textsf{ptde}}^*, m_1)$$, then $$B $$ gives the view of $$\textsf{Game} _{5}$$ (conditioned on $$b = 1$$) to $$A $$. Otherwise, if $$c^*:= c_0'$$ is generated as $$\Sigma .\textsf{Enc} (k_{\textsf{ptde}}^*, m_0)$$, then the view is of $$\textsf{Game} _{6}$$. Therefore, if $$A $$ can distinguish between the two games with non-negligible advantage, then $$B $$ must also have non-negligible advantage against the puncturable tag-based deterministic encryption security game. $$\square $$

This concludes the proof of Theorem 2.

### Extending Supported Predicates

For our generic construction, it is easily possible to extend it from supporting the equality test predicate (i.e., tags) to more powerful predicates, i.e., an access control mechanism known from ABE in the terminology of [9].

Let us follow the notation of Gorbunov et al. [38], who construct ABE for any circuit of arbitrary polynomial size. Thus, let $\mathsf ind$ be an $\ell$ bit public index (used for encryption) and $\textbf{P}$ a Boolean predicate (associated with secret keys) and decryption should only work if $\textbf{P}(\textsf{ind})=1$. Now, we can simply associate function keys with more expressive predicates $\textbf{P}$ (encode them into PKey) instead of tags and use as public tags for the PTDE scheme the public index $\mathsf ind$ (i.e., the attributes). In the decryption circuit $P_{f,\textbf{P}}$, one simply checks if for label $\mathsf ind$ and hard-coded $\textbf{P}$ it holds that $\textbf{P}(\textsf{ind})=1$.

Switching the public index in a ciphertext from $\mathsf ind$ to some $\mathsf ind'$, i.e., change the attributes in the ciphertext, can simply be done by viewing the public indices as the tags in the current solution. Now, this represents a generalization of our generic construction where we only have the equality predicate $\textbf{P}_t({\hat{t}})=1$ if and only if $t={\hat{t}}$.

## Lattice-Based CUFE Construction for Inner Products

After recalling the syntax and properties of the main sampling algorithms used in lattice-based constructions, we will build a CUFE scheme for inner-products from the LWE assumption in the random-oracle model in this section. For a further exposition of lattice preliminaries, we refer the reader to Appendix A.2.

### Lattice Definitions and Algorithms

For any matrix $\textbf{A}\in \mathbb {Z} _q^{n\times m}$, we define the orthogonal *q*-ary lattice of $\textbf{A}$ as $\Lambda _q^\perp (\textbf{A}):=\{\vec {u}\in \mathbb {Z} ^m:\textbf{A}\vec {u}=\vec {0} \mod q \}$.

The normal Gaussian distribution of mean 0 and variance $\sigma ^2$ is the distribution on $\mathbb {R}$ with probability density function $\frac{1}{\sigma \sqrt{2\pi }}\frac{1}{e^{x^2/(2\sigma ^2)}}$. The lattice Gaussian distribution with support a lattice $\Lambda \subseteq \mathbb {Z} ^m$, standard deviation $\sigma$ and centered at $\vec {c}\in \mathbb {Z} ^m$, is defined as:

$$\begin{aligned} \text {for all }\vec {y}\in \Lambda : \mathcal {D}_{\Lambda , \sigma , \vec {c}}(\vec {y})=\frac{e^{-\pi \Vert \vec {y}-\vec {c}\Vert ^2/\sigma ^2}}{\sum _{\vec {x}\in \Lambda }e^{-\pi \Vert \vec {x}-\vec {c}\Vert ^2/\sigma ^2}} \end{aligned}$$

The following algorithms will be used in lattice construction, and their properties needed in the security proof.

#### Lemma 9

([37] Preimage Sampleable Functions) For any prime $q=poly(n)$, any $m\ge 5n\log q$, and any $s\ge m^{2.5}\omega (\sqrt{\log m})$, it holds that there exist PPT algorithms $\textsf{TrapGen}$, $\textsf{SampleD}$, $\textsf{SamplePre}$ such that:

1. $\textsf{TrapGen}$ computes $(\textbf{A}, \textbf{T})\leftarrow \textsf{TrapGen}(1^n, 1^m)$, where $\textbf{A}\in \mathbb {Z} _q^{n\times m}$ is statistically close to uniform and $\textbf{T}\subset \Lambda _q^\perp (\textbf{A})$ is a basis with $\Vert \widetilde{\textbf{T}}\Vert \le m^{2.5}$. The matrix $\textbf{A}$ (and *q*) is public, while the good basis $\textbf{T}$ is the trapdoor.
2. $\textsf{SampleD}$ samples matrices $\textbf{Z}'$ from $\mathcal {D}_{\mathbb {Z} ^{m\times m}, s}$,
3. The trapdoor inversion algorithm $\textsf{SamplePre}(\textbf{A}, \textbf{T}, \textbf{D}, s)$, for $\textbf{D}\in \mathbb {Z} _q^{n\times m}$, outputs a matrix $\textbf{Z}\in \mathbb {Z} ^{m\times m}$ such that $\textbf{A}\textbf{Z}=\textbf{D}$.

In addition, it holds that the following distributions $D_1$, $D_2$ are statistically close:

$$\begin{aligned} D_1=(\textbf{A}, \textbf{Z}, \textbf{D}), \text { s.t. } (\textbf{A}, \textbf{T})\leftarrow \textsf{TrapGen}(1^n,1^m), \textbf{D}\leftarrow \mathbb {Z} _q^{n\times m}, \\ \textbf{Z}\leftarrow \textsf{SamplePre}(\textbf{A}, \textbf{T}, \textbf{D}, s), \\ D_2=(\textbf{A}, \textbf{Z}', \textbf{A}\textbf{Z}'), \text { where } \textbf{A}\leftarrow \mathbb {Z} _q^{n\times m}, \textbf{Z}'\leftarrow \mathcal {D}_{\mathbb {Z} ^{m\times m}, s}. \end{aligned}$$

#### Theorem 3

([1] SampleLeft) Let $q>2$, full rank $\textbf{A}, \textbf{B}\in \mathbb {Z} _q^{n\times m}$ with $m>n$, a basis $\textbf{T}_{\textbf{A}}$ of $\Lambda _q^\perp (\textbf{A})$, a matrix $\textbf{D}\in \mathbb {Z} _q^{n\times m}$ and $\sigma >\Vert \widetilde{\textbf{T}}_{\textbf{A}}\Vert \cdot \omega (\sqrt{\log m})$. Then there exists PPT algorithm. $\textsf{SampleLeft}(\textbf{A}, \textbf{T}_{\textbf{A}}, \textbf{B}, \textbf{D}, \sigma )$ that outputs a matrix $\textbf{X}\in \mathbb {Z} ^{2m\times m}$, distributed statistically close to $\mathcal {D}_{\Lambda _q^{\textbf{D}}(\textbf{A}|\textbf{B}), \sigma }$.

### Lattice Construction

**Fig. 2:**
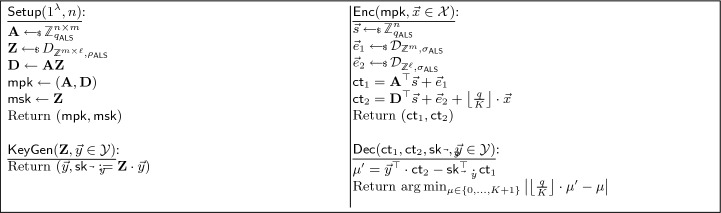
Inner-product functional encryption scheme $\textsf{ALS}$, with parameters as in [9]

We are building on the work of Abdalla et al. [9], who gave the first constructions, one in the standard model (SM) and one in the random-oracle model (ROM), of a lattice-based identity-based IPFE scheme, and proved their security^15^ under the $\text {LWE}_{q,\alpha ,n}$ assumption (Definition 10). Their constructions are in turn based on the IPFE scheme of Agrawal et al. [15], ${\textsf{ALS}}$, described in Fig. 2.

**Fig. 3:**
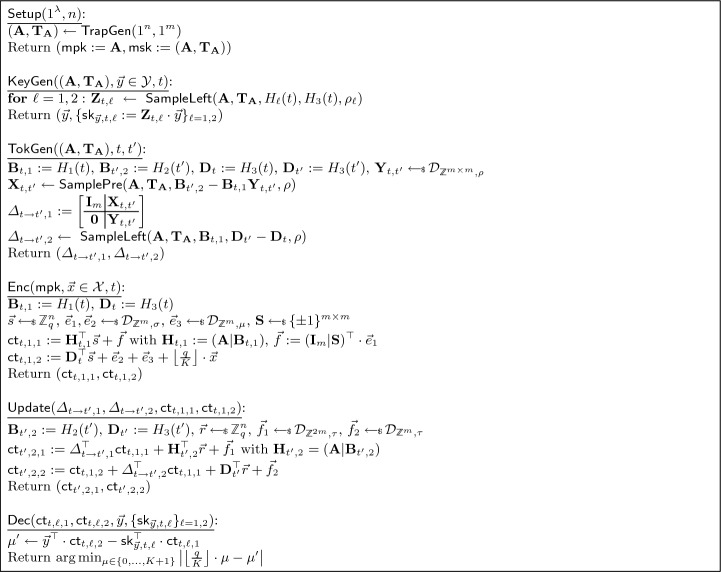
Lattice-based Ciphertext-Updatable IPFE scheme

In our construction, we start from the ROM scheme of Abdalla et al. [9] and enhanced their design in order to allow distinguishing fresh and updated ciphertexts. To prove its security, we rely on the programmability of random oracles $H_1, H_2, H_3:\mathcal {T}\rightarrow \mathbb {Z}_q^{n\times m}$, where $\mathcal {T}$ is the tag space. Notice that programmability of random oracles is required in the security proof to simulate the new supported functionality, i.e., updating ciphertexts. Thus, even though our construction is only proved secure in the ROM, it also supports a richer class of functionalities than previous works. Our lattice-based CUFE construction is described in Fig. 3. Dimensions of matrices involved in the construction are presented in Table 1.

**Table 1:** Matrices, vectors, and respective dimensions used in the construction

$\textbf{A}$		$\mathbb {Z}_q^{n\times m}$	$\textbf{X}_{t,t '}$		$\mathbb {Z}^{m\times m}$
$\textbf{T}_{\textbf{A}}$		$\mathbb {Z}^{m\times m}$	$\textbf{Y}_{t,t '}$		$\mathbb {Z}^{m\times m}$
$\textbf{B}_{t,1}$		$\mathbb {Z}_q^{n\times m}$	$\vec {s}$		$\mathbb {Z}_q^{n}$
$\textbf{B}_{t,2}$		$\mathbb {Z}_q^{n\times m}$	$\vec {e}_1$		$\mathbb {Z}^{m}$
$\textbf{D}_{t}$		$\mathbb {Z}_q^{n\times m}$	$\vec {e}_2$		$\mathbb {Z}^{m}$
$\Delta _{t \rightarrow t ',1}$		$\mathbb {Z}^{2m\times 2m}$	$\vec {e}_3$		$\mathbb {Z}^{m}$
$\Delta _{t \rightarrow t ',2}$		$\mathbb {Z}^{2m\times m}$	$\textbf{S}$		$\{\pm 1\}^{m\times m}$
${\textsf{ct}}_{t,1,1}$		$\mathbb {Z}_q^{2m}$	$\vec {f}_1$		$\mathbb {Z}^{2m}$
${\textsf{ct}}_{t,1,2}$		$\mathbb {Z}_q^{m}$	$\vec {f}_2$		$\mathbb {Z}^{m}$
${\textsf{ct}}_{t,2,1}$		$\mathbb {Z}_q^{2m}$	$\vec {f}$		$\mathbb {Z}^{m}$
${\textsf{ct}}_{t,2,2}$		$\mathbb {Z}_q^{m}$	$\vec {x}$		$\{0,\dots ,P\}^m$
$\textbf{Z}_{t,1}$		$\mathbb {Z}^{2m\times m}$	$\vec {y}$		$\{0,\dots ,V\}^m$
$\textbf{Z}_{t,2}$		$\mathbb {Z}^{2m\times m}$	$\langle \vec {y},\vec {x}\rangle$		$\{0,\dots ,mPV\}$

The first component of the ciphertext, $${\textsf{ct}}_{t,1,1}$$, depends on the tag $$t $$ but not on the message. The second component, $${\textsf{ct}}_{t,1,2}$$, on the other hand, depends on the message $$\vec {x}$$ to be encrypted. The two components are intertwined by the shared randomness $$\vec {s}\in \mathbb {Z}_q^n$$. In order to update ciphertexts, it is therefore necessary to update the two parts of a given ciphertext to the prescribed new tag, while preserving the common randomness, the underlying plaintext, and, at the same time, without increasing the error term too much. Latter would prevent correct decryption of updated ciphertexts. This can be done using techniques inspired by [21, 33]. Moreover, since the randomness is given by uniform vector in $$\mathbb {Z}_q^n$$ and the encryption scheme is additively homomorphic, ciphertexts can be easily re-randomized.

To update a ciphertext from $$t $$ to $$t '$$, we want to produce a $$2m\times 2m$$ matrix $$\Delta _{t \rightarrow t ',1}$$ over $$\mathbb {Z}$$ and a $$2m\times m$$ matrix $$\Delta _{t \rightarrow t ',2}$$ over $$\mathbb {Z}$$, with $$\Delta _{t \rightarrow t ',2}\leftarrow \mathcal {D}_{\mathbb {Z} ^{2m\times m}, \rho }$$. $$\Delta _{t \rightarrow t ',1}$$ has the form

$$\begin{aligned} \Delta _{t \rightarrow t ',1}:= \left[ \begin{array}{c|c} \textbf{I}_m &{} \textbf{X}_{t,t '} \\ \hline \textbf{0} &{} \textbf{Y}_{t,t '} \end{array} \right] , \end{aligned}$$

with $\textbf{X}_{t,t '}, \textbf{Y}_{t,t '}\leftarrow \mathcal {D}_{\mathbb {Z} ^{m\times m}, \rho }$. $\Delta _{t \rightarrow t ',1}$ and $\Delta _{t \rightarrow t ',2}$ are additionally conditioned on

$$\begin{aligned} \textbf{H}_{t,1}\cdot \Delta _{t \rightarrow t ',1}=\textbf{H}_{t ',2}, \quad \text { and }\quad \textbf{H}_{t,1}\cdot \Delta _{t \rightarrow t ',2}=\textbf{D}_{t '}-\textbf{D}_{t}. \end{aligned}$$

In the real game the matrix $\Delta _{t \rightarrow t ',1}$ and $\Delta _{t \rightarrow t ',2}$ will be produced using the trapdoor $\textbf{T}_{\textbf{A}}$, i.e., $\textbf{Y}_{t,t '}$ will be sampled from $\mathcal {D}_{\mathbb {Z} ^{m\times m}, \rho }$, $\textbf{X}_{t,t '}$ using $\textsf{SamplePre}(\textbf{A}, \textbf{T}_{\textbf{A}},\textbf{B}_{t ',2}-\textbf{B}_{t,1}\textbf{Y}_{t,t '},\rho )$, where $\textbf{H}_{t ',2}=(\textbf{A}|\textbf{B}_{t ',2})$, and $\Delta _{t \rightarrow t ',2}$ using $\textsf{SampleLeft}(\textbf{A},\textbf{T}_{\textbf{A}},\textbf{B}_{t,1},\textbf{D}_{t '}-\textbf{D}_{t},\rho )$.

Vice versa, in the security proof, we will leverage on the programmability of the random oracles $$H_1$$, $$H_2$$, and $$H_3$$: whenever the source tag $$t $$ equals the challenge tag $$t ^*$$, $$\textbf{X}_{t,t '}$$, $$\textbf{Y}_{t,t '}$$, and $$\Delta _{t \rightarrow t ',2}$$ will be sampled from the appropriate distributions, $$H_2(t ')=\textbf{B}_{t ',2}$$ will be set to equal $$\textbf{A}\textbf{X}_{t,t '}+\textbf{B}_{t,1}\textbf{Y}_{t,t '}$$, and $$H_3(t ')=\textbf{D}_{t '}$$ to $$\textbf{H}_{t,1}\cdot \Delta _{t \rightarrow t ',2}+\textbf{D}_{t}$$. For all other pair of tags, $$t, t '$$, the token $$(\Delta _{t \rightarrow t ',1},\Delta _{t \rightarrow t ',2})$$ is produced using the trapdoor of $$H_1(t)=\textbf{B}_{t,1}$$: The matrix $$\textbf{B}_{t,1}$$ will be produced using the $$\textsf{TrapGen}$$ algorithm, and the update token will be produced using such trapdoor.

To update a ciphertext $({\textsf{ct}}_{t,1,1},{\textsf{ct}}_{t,2,2})$, given the appropriate token $(\Delta _{t \rightarrow t ',1},\Delta _{t \rightarrow t ',2})$, fresh randomness $\vec {r}\leftarrow \mathbb {Z} _q^n$ and noises $\vec {f}_1\leftarrow \mathcal {D}_{\mathbb {Z} ^{2\,m}, \tau }$,$\vec {f}_2\leftarrow \mathcal {D}_{\mathbb {Z} ^{m}, \tau }$ are sampled and the new ciphertext $({\textsf{ct}}_{t ',2,1},{\textsf{ct}}_{t ',2,2})$ is computed as

$$\begin{aligned} {\textsf{ct}}_{t ',2,1}&:=\Delta _{t \rightarrow t ',1}^\top {\textsf{ct}}_{t,1,1}+\textbf{H}_{t ',2}^\top \vec {r}+\vec {f}_1\text {,} \\ {\textsf{ct}}_{t ',2,2}&:={\textsf{ct}}_{t,1,2}+\Delta _{t \rightarrow t ',2}^\top {\textsf{ct}}_{t,1,1}+\textbf{D}_{t '}^\top \vec {r}+\vec {f}_2\text {.} \end{aligned}$$

The functional secret keys, $\{sk _{t, \ell , \vec {y}}\}_{\ell =1,2}$, can be produced as follows:

1. for the challenge tag $t ^*$: for $\ell =1$, using the ALS challenger, and for $\ell =2$, using the trapdoor of $\textbf{B}_{t ^*,2}$.
2. for tags, $t \ne t ^*$, for which no update token of the form $(\Delta _{t ^*\rightarrow t,1},\Delta _{t ^*\rightarrow t,2})$ was queried but to which the challenge ciphertext was updated: using the trapdoor of $\textbf{B}_{t,1}$, or again the ALS challenger for $\ell =2$.
3. for all other tags: using the trapdoor of $\textbf{B}_{t,\ell }$ for $\ell =1,2$.

*Parameters and Correctness* In our construction, ciphertexts encode vectors $$\vec {x}\in \{0,\dots ,P\}^m$$ under a tag $$t $$. Secret keys correspond to a tag $$t $$ and a vector $$\vec {y}\in \{0,\dots ,V\}^m$$. When tags match, our scheme decrypts the bounded inner-product $$\langle \vec {x}, \vec {y}\rangle \in \{0, \dots , K\}$$, where $$K=mPV$$. Moreover, our scheme parameters must satisfy the following bounds:

- $$m\ge 6 n \log q$$ (required by $$\textsf{TrapGen}$$),
- $\alpha q > 2\sqrt{n}$ (required by hardness of LWE).
- $$\rho =\rho _1=\rho _{\text {ALS}}\ge m^{2.5}\cdot \omega (\sqrt{\log m})$$ (required by $$\textsf{SamplePre}$$),
- $\rho _2\ge m\rho \cdot \lambda ^{\omega (1)}$ (required in the security proof for the indistinguishability of function keys),
- $$\sigma =\sigma _{\text {ALS}}$$,
- $\textsf{NoiseGen}$: the spectral norm of $\textbf{S}_{t ^*}$ can be upper bounded (by using the Frobenius norm) by *m*. Using Lemma 15, $s_1(\textbf{Z}_{t ^*})\le 3C \rho \sqrt{m}$, which implies $\mu \ge 3C \rho m^{1.5}$,
- $\tau \ge \sqrt{m}(\sigma +\mu +2\sqrt{2}\rho \sigma m^{1.5}C')\lambda ^{\omega (1)}$ (require in the security proof for the indistinguishability of updated honest ciphertexts) and $\tau \ge (\sigma \sqrt{m}+\sigma \rho _2 m^{1.5}+\sqrt{2}m^2\sigma \rho _2 C')\lambda ^{\omega (1)}$ (for the indistinguishability of updates of the challenge ciphertext). Thus, we set $\tau \ge \max \{\sqrt{m}(\sigma +\mu +2\sqrt{2}\rho \sigma m^{1.5}C'),(\sigma \sqrt{m}+\sigma \rho _2 m^{1.5}+\sqrt{2}m^2\sigma \rho _2 C')\}\cdot \lambda ^{\omega (1)}$,
- $q>2KVm(\sigma +\mu +\tau +12\sqrt{2}C'm^{2.5}\rho _2(\rho \sigma +\tau ))$ (required for successful decryption of updated ciphertexts),

#### Lemma 10

(Correctness) For $q>2KVm(\sigma +\mu +\tau +12\sqrt{2}C'm^{2.5}\rho _2(\rho \sigma +\tau ))$, the decryption of (updated) ciphertexts from the scheme in Fig. 3 is, w.h.p., correct.

#### Proof

The correct decryption of fresh ciphertexts follows directly from the correctness of the Abdalla et al. [9] construction. On the other hand, an updated ciphertext has the following form:

$$\begin{aligned} \begin{aligned} {\textsf{ct}}_{t ',2,1}&:=\Delta _{t \rightarrow t ',1}^\top {\textsf{ct}}_{t,1,1}+\textbf{H}_{t ',2}^\top \vec {r}+\vec {f}_1\\&=\textbf{H}_{t ',2}^\top (\vec {s}+\vec {r})+\Delta _{t \rightarrow t ',1}^\top \vec {f}+\vec {f}_1 \text {, and} \\ {\textsf{ct}}_{t ',2,2}&:={\textsf{ct}}_{t,1,2}+\Delta _{t \rightarrow t ',2}^\top {\textsf{ct}}_{t,1,1}+\textbf{D}_{t '}^\top \vec {r}+\vec {f}_2\\&=\textbf{D}_{t '}^\top (\vec {s}+\vec {r})+\vec {e}_2+\vec {e}_3+\Delta _{t \rightarrow t ',2}^\top \vec {f}+\vec {f}_2+\left\lfloor {\frac{q}{K}}\right\rfloor \cdot \vec {x}. \end{aligned} \end{aligned}$$

Therefore, during decryption of updated ciphertexts, one obtains:

$$\begin{aligned} \begin{aligned} \mu '&=\vec {y}^\top \cdot {\textsf{ct}}_{t,2,2}-{\textsf{sk}}_{t,2,\vec {y}}^\top \cdot {\textsf{ct}}_{t,2,1}\\&=\left\lfloor {\frac{q}{K}}\right\rfloor \langle \vec {y},\vec {x}\rangle +\underbrace{\vec {y}^\top (\vec {e}_2+\vec {e}_3+\Delta _{t \rightarrow t ',2}^\top \vec {f}+\vec {f}_2)-\vec {y}^\top \textbf{Z}^\top _{t ',2}(\Delta _{t \rightarrow t ',1}^\top \vec {f}+\vec {f}_1)}_\text {error terms}, \end{aligned} \end{aligned}$$

where we have used the fact that $\textbf{H}_{t ',2}\cdot \textbf{Z}_{t ',2}=\textbf{D}_{t '}$. This decrypts correctly as long as the error terms obtained

$$\begin{aligned} \vec {y}^\top (\vec {e}_2+\vec {e}_3+\Delta _{t \rightarrow t ',2}^\top \vec {f}+\vec {f}_2-\textbf{Z}^\top _{t ',2}(\Delta _{t \rightarrow t ',1}^\top \vec {f}+\vec {f}_1)), \end{aligned}$$

are small compared to *q*/*K*. Since $\Delta _{t,t ',1}\in \mathbb {Z}^{2m\times 2m}$, and $\Delta _{t,t ',2},\textbf{Z}_{t ',2}\in \mathbb {Z}^{2\,m\times m}$ are sampled via the $\textsf{SamplePre}$ algorithm with parameter $\rho$ and $\rho _2$, respectively, by Lemma 11, we know that $\Vert \textbf{Z}_{t ',2}\Vert \le 2m\cdot \rho _2$, $\Vert \Delta _{t \rightarrow t ',1} \Vert \le 2m\cdot \rho$, and $\Vert \Delta _{t \rightarrow t ',2} \Vert \le \sqrt{2}\cdot m\cdot \rho$, as long as $\rho ,\rho _2\ge m^{2.5}\omega (\sqrt{\log n})$. Using again Lemma 11 and Lemma 14, we can also deduce that $\Vert \vec {e}_1\Vert ,\Vert \vec {e}_2\Vert \le \sigma \sqrt{m}$, $\Vert \vec {e}_3\Vert \le \mu \sqrt{m}$, $\Vert \vec {f}\Vert \le C'\sigma \sqrt{2}m$ and $\Vert \vec {f}_1\Vert \le \tau \sqrt{2\,m}$,$\Vert \vec {f}_2\Vert \le \tau \sqrt{m}$, as long as $\sigma ,\mu ,\tau \ge \omega (\sqrt{\log n})$. Therefore, $\Vert \Delta _{t \rightarrow t ',1}^\top \vec {f}\Vert \le 2\sqrt{2}C'm^2\rho \sigma$, $\Vert \Delta _{t \rightarrow t ',2}^\top \vec {f}\Vert \le 2C'm^2\rho \sigma$, and $\Vert \textbf{Z}^\top _{t ',2}(\Delta _{t \rightarrow t ',1}^\top \vec {f}+\vec {f}_1)\Vert \le 2\,m\rho _2(2\sqrt{2}C'm^2\rho \sigma +\sqrt{2\,m}\tau )$. Since, $\Vert \vec {y}\Vert \le V\sqrt{m}$, the final error term is upper bounded by $V\sqrt{m}\cdot (\sigma \sqrt{m}+\mu \sqrt{m}+2C'm^2\rho \sigma +\tau \sqrt{m} +2\,m\rho _2(2\sqrt{2}C'm^2\rho \sigma +\sqrt{2\,m}\tau ))$. For decryption to succeed, we want that the error term is smaller than $\frac{q}{2K}$, which implies:

$$\begin{aligned} \begin{aligned} q&>2K V\sqrt{m}\cdot (\sigma \sqrt{m}+\mu \sqrt{m}+2C'm^2\rho \sigma +\tau \sqrt{m} +2m\rho _2(2\sqrt{2}C'm^2\rho \sigma +\sqrt{2m}\tau ))\\&>2KVm(\sigma +\mu +\tau +12\sqrt{2}C'm^{2.5}\rho _2(\rho \sigma +\tau )). \square \end{aligned} \end{aligned}$$

*Security Proof* We now show that the adaptive security of our CUFE construction follows from the security of the ALS scheme. In order to do so, we, however, have to make the following restrictions regarding the validity of the adversary in the $\mathsf {IND\text {-}CUFE\text {-}CPA}$ experiment:

1. If $(\cdot ,t ^*,t ')\in {\mathcal{C}\mathcal{T}}$, then there is no $(f,t ')\in \mathcal {K}$,
2. For any $t\in \mathcal {T}$, the number of $\textsf{CorTokGen}$ oracle queries, on input $(t,\cdot )$, is bounded by a constant,^16^
3. The number of $\textsf{HonUpdate}$ oracle queries, on input $(\cdot ,\cdot ,0,\cdot )$, is bounded by a constant.

The first restriction is due to limitations in our current proof techniques: Given $t'\in \mathcal {T}$, $t'\ne t^*$, the reduction can either simulate $\Delta _{t^*\rightarrow t'}$, or $sk _{f,t'}$, for any arbitrary *f*. Since $\textsf{CorTokGen}$ requires generating $\Delta _{t^*\rightarrow t'}$, the reduction would not be able to simulate $sk _{f,t'}$ as well. The last two restrictions are instead due to the security loss that the guessing strategy would otherwise lead to as the target tags of tokens, where the source tag is the challenge one, and challenge update queries made have to be guessed in advance. Since the proof is in the ROM, these guesses are not over the entire tag space $\mathcal {T}$, which can be unbounded, but over the indices of the RO queries, which are bounded by a polynomial in the security parameter as the adversary needs to be efficient. As long as the number of $\textsf{CorTokGen}$-oracle queries per given source tag, and $\textsf{HonUpdate}$-oracle queries on input the challenge ciphertext, is constant, the security loss will be polynomially bounded. We will make this assumption in Theorem 4. This result can also be rephrased in the following terms: If one maintains a “recording graph” that has a node for each tag queried to the RO, and whose edges are derived from the tokens and challenge updates issued to the adversary, then the loss is given by $n^{\delta }$, where *n* is the number of nodes in the graph, and $\delta$ is the outer degree of the graph. This result is similar to the one obtained by Fuchsbauer et al. [32], who show how to generically obtain proxy re-encryption (PRE) schemes secure against adversaries that can adaptively corrupt users from PRE schemes secure against adversaries that cannot make adaptive user corruptions. They do so by reducing the simulation in the security proof to a pebbling game on the graph “underlying” the security game [39]. We believe that any improvement to the results of Fuchsbauer et al. [32] and Jafargholi et al. [39] could also offer useful insights on how to overcome the current limitation of our construction.

#### Theorem 4

(Security) Let $\lambda$ be the security parameter. Fix parameters *q*, *n*, *m*, $\alpha$, $\sigma$, $\rho$, $\rho _1$, $\rho _2$, $\mu$, and $\tau$ as above. Then, under the above restrictions on the adversary, the $\textsf{CUFE}$ scheme described in Fig 3 is adaptive $\mathsf {IND\text {-}CUFE\text {-}CPA}$-secure if the ALS-IPFE scheme [15] is ${\textsf{AD}}$-$\textsf{IND}$ secure.

#### Proof

We proceed in a series of hybrids, consider $$\mathcal {A}$$ to be a PPT adversary, and $$\lambda $$ to be the security parameter. We denote by $$\textbf{Adv}_{\text {Game}_i}(\mathcal {A})$$ the advantage of $$\mathcal {A}$$ in Game *i*. Let $$Q_{\text {h}}$$ be the number of random-oracle queries made by the adversary, $$Q_{\text {t}}$$ be the maximum number of $$\textsf{TokGen}$$-oracle queries of the form $$(t,t_i)$$ for any fixed tag $$t $$, and $$Q_{\text {u}}$$ be the maximum number of $$\textsf{Update}$$-oracle queries on input the challenge ciphertext. We will assume, without loss of generality, that any adversary making key generation queries of the form $$(\vec {y},t)$$, update queries of the form $$(t,t ',\cdot ,\cdot )$$, or token generation queries of the form $$(t,t ')$$ will first query the random oracle *H* on $$t $$ and $$t '$$. (We can make this assumption because for every adversary $$\mathcal {A}$$, we can compile it into an adversary $$\mathcal {A}'$$ that exhibits this behavior.)

$\textsf{Game}_{0}$. This is the original $\mathsf {IND\text {-}CUFE\text {-}CPA}$ game.

$\textsf{Game}_{1}$. This is the same as previous game, except that we guess the tag $t ^*$ which will be used for the challenge messages. Instead of guessing directly $t ^*$ among the set of tags $\mathcal {T}$, which would incur an exponential loss, we guess the index of the random-oracle query in which the adversary queries *H* to get $\textbf{H}_{t ^*,1}$ and $\textbf{D}_{t ^*}$. If the guess is incorrect, we abort. This results in a $\frac{1}{Q_{\text {h}}}$ security loss.

$\textsf{Game}_{2}$. This is the same as previous game, except that we guess for which tags $t '$ the adversary will query an update token of the form $(\Delta _{t ^*\rightarrow t ',1},\Delta _{t ^*\rightarrow t ',2})$. If the guess was incorrect, we abort. As above, instead of guessing directly the tag $t '$ among the set of tags $\mathcal {T}$, which would incur an exponential loss, we guess the indices of the random-oracle query in which the adversary queries *H* to get $\textbf{H}_{t ',2}$ and $\textbf{D}_{t '}$. This will result in a $$\left( {\begin{array}{c}Q_{\text {h}} - 1\\ Q_{\text {t}}\end{array}}\right) ^{-1}$$ security loss.

$\textsf{Game}_{3}$. This is the same as previous game, except that we guess for which tags $t '$ the adversary will query the $\textsf{Update}$-oracle on input the challenge ciphertext. As above, instead of guessing directly the tag $t '$ among the set of tags $\mathcal {T}$, which would incur in an exponential loss, we guess the indices of the random-oracle query in which the adversary queries *H* to get $\textbf{H}_{t ',2}$ and $\textbf{D}_{t '}$. If the guess is incorrect, we abort. This results in a $$\left( {\begin{array}{c}Q_\text {h}-Q_\text {t}-1\\ Q_{\text {u}}\end{array}}\right) ^{-1}$$ security loss.

From now on, let $\mathcal {H}=\{t _1,\cdots ,t _{Q_{\text {h}}}\}$ be the list of random-oracle queries made by the adversary. Let $i^*\in [Q_{\text {h}}]$ be the index of the query corresponding to the challenge tag, i.e., $t _{i^*}=t ^*$. Let $\mathcal{Q}\mathcal{T}$ be the list of indices $\{i_k\}_{k\le Q_{\text {t}}}$ for which the adversary will query an update token from the challenge tag $t ^*$, and let $\mathcal{Q}\mathcal{U}$ be the list of indices $\{j_k\}_{k\le Q_{\text {u}}}$ for which the adversary will query the $\textsf{Update}$-oracle for a ciphertext encrypted under the challenge tag $t ^*$.

$\textsf{Game}_{4}$. This is the same as previous game, except for the following modifications. For each of $i_k\in \mathcal{Q}\mathcal{T}$, we sample $\textbf{X}_{t ^*,t _{i_k}}, \textbf{Y}_{t ^*,t _{i_k}}\leftarrow \mathcal {D}_{\mathbb {Z} ^{m\times m}, \rho }$, and $\Delta _{t ^*\rightarrow t,2}\leftarrow \mathcal {D}_{\mathbb {Z} ^{2\,m\times m}, \rho }$. Then, we set $H_2(t _{i_k}):=\textbf{B}_{t _{i_k},2}:=\textbf{A}\textbf{X}_{t ^*,t _{i_k}}+\textbf{B}_{t ^*,1}\textbf{Y}_{t ^*,t _{i_k}}$ and $H_3(t _{i_k}):=\textbf{D}_{t _{i_k}}=\textbf{H}_{t ^*,1}\Delta _{t ^*\rightarrow t,2}+\textbf{D}_{t ^*}$. When the adversary queries the $\textsf{CorTokGen}$ oracle on input $(t,t ')$ we return

$$\begin{aligned} \Delta _{t ^*\rightarrow t,1}:= \left[ \begin{array}{c|c} \textbf{I}_m &{} \textbf{X}_{t ^*,t _{i_k}} \\ \hline \textbf{0} &{} \textbf{Y}_{t ^*,t _{i_k}} \end{array} \right] \quad \text { and }\quad \Delta _{t ^*\rightarrow t,2}, \end{aligned}$$

to the adversary. The rest of the game is as before. By Lemma 9, each of the token $(\Delta _{t ^*\rightarrow t,1},\Delta _{t ^*\rightarrow t,2})$ is distributed statistically close to the previous game.

$\textsf{Game}_{5}$. This is the same as previous game, except for the following modifications. For all $i\in [Q_{\text {h}}], i\ne i^*$, we sample $(\textbf{B}_{t _i,1},\textbf{T}_{\textbf{B}_{t _i,1}})\leftarrow \textsf{TrapGen}(1^n,1^m)$ and set $H_1(t _i):=\textbf{B}_{t _i,1}$. Whenever the adversary makes a query to the $\textsf{CorTokGen}$ oracle of the form $(t _i,t)$, we reply using $\textbf{T}_{\textbf{B}_{t _i, 1}}$ instead of $\textbf{T}_{\textbf{A}}$:

- sample $\textbf{X}_{t _i,t}\leftarrow \mathcal {D}_{\mathbb {Z} ^{m\times m}, \rho }$, run $\textbf{Y}_{t ',t}\leftarrow \textsf{SamplePre}(\textbf{B}_{t _i,1}, \textbf{T}_{\textbf{B}_{t _i,1}},\textbf{B}_{t,2}-\textbf{A}\textbf{X}_{t _i,t},\rho )$, along with $\textbf{R}_{t _i\rightarrow t,2}\leftarrow \textsf{SampleLeft}(\textbf{B}_{t _i,1}, \textbf{T}_{\textbf{B}_{t _i,1}},\textbf{A},\textbf{D}_{t}-\textbf{D}_{t _i},\rho )$. Return
  $$\begin{aligned} \Delta _{t _i\rightarrow t,1}:= \left[ \begin{array}{c|c} \textbf{I}_m &{} \textbf{X}_{t ',t} \\ \hline \textbf{0} &{} \textbf{Y}_{t ',t} \end{array} \right] \quad \text { and }\quad \Delta _{t _i\rightarrow t,2}:=\left[ \begin{array}{c|c} \textbf{0}&{} \textbf{I}_{m} \\ \hline \textbf{I}_{m} &{} \textbf{0} \end{array} \right] \cdot \textbf{R}_{t _i\rightarrow t,2}. \end{aligned}$$

The rest of the game is as before. Notice that, by the invariance under permutation of the Gaussian distribution, we have that $\Delta _{t _i\rightarrow t,2}\leftarrow \mathcal {D}_{\mathbb {Z} ^{2\,m\times m}, \rho }$. Moreover,

$$\begin{aligned} \textbf{H}_{t _i,1}\Delta _{t _i\rightarrow t,2}=(\textbf{A}|\textbf{B}_{t _i,1})\left[ \begin{array}{c|c} \textbf{0}&{} \textbf{I}_{m} \\ \hline \textbf{I}_{m} &{} \textbf{0} \end{array} \right] \cdot \textbf{R}_{t _i\rightarrow t,2}=(\textbf{B}_{t _i,1}|\textbf{A})\textbf{R}_{t _i\rightarrow t,2}=\textbf{D}_{t}-\textbf{D}_{t _i}, \end{aligned}$$

as expected. Applying again Lemma 9, we also obtain that the distribution of $\textsf{CorTokGen}$-oracle’s replies is statistically close to that of $\textsf{Game}_{4}$.

$\textsf{Game}_{6}$. This is the same as previous game, except for the following modifications. Now, for all $i\not \in \{i_1,\dots ,i_{Q_{\text {t}}}\}\cup \{j_1,\dots ,j_{Q_{\text {u}}}\}$, we sample $(\textbf{B}_{t _i,2},\textbf{T}_{\textbf{B}_{t _i,2}})\leftarrow \textsf{TrapGen}(1^n,1^m)$ and set $H_2(t _i):=\textbf{B}_{t _i,2}$. Whenever the adversary makes a query to the $\textsf{KeyGen}'$ oracle of the form $(t _i,\vec {y})$, with $i\not \in \{i_1,\dots ,i_{Q_{\text {t}}}\}\cup \{j_1,\dots ,j_{Q_{\text {u}}}\}\cup \{i^*\}$, we reply using $\textbf{T}_{\textbf{B}_{t _i,1}}$ and $\textbf{T}_{\textbf{B}_{t _i,2}}$ instead of $\textbf{T}_{\textbf{A}}$ (recall that $\textbf{T}_{\textbf{B}_{t _i,1}}$ was already introduced in the previous game for all $i\ne i^*$):

- for $\ell =1,2$, run $\textsf{SampleLeft}(\textbf{B}_{t _i,\ell }, \textbf{T}_{\textbf{B}_{t _i,\ell }},\textbf{A},\textbf{D}_{t _i},\rho _\ell )$ to obtain $\textbf{R}_{t _i,\ell }$. Return
  $$\begin{aligned} \textbf{Z}_{t _i,\ell }:= \left[ \begin{array}{c|c} \textbf{0}&{} \textbf{I}_{m} \\ \hline \textbf{I}_{m} &{} \textbf{0} \end{array} \right] \cdot \textbf{R}_{t _i,\ell }. \end{aligned}$$

The rest of the game is as before. Notice that, by the invariance under permutation of the Gaussian distribution, we have that $\textbf{Z}_{t _i,\ell }\leftarrow \mathcal {D}_{\mathbb {Z} ^{2\,m\times 2\,m}, \rho _\ell }$. Moreover,

$$\begin{aligned} \textbf{H}_{t _i,\ell _i}\textbf{Z}_{t _i,\ell _i}=(\textbf{A}|\textbf{B}_{t _i,\ell _i})\left[ \begin{array}{c|c} \textbf{0}&{} \textbf{I}_{m} \\ \hline \textbf{I}_{m} &{} \textbf{0} \end{array} \right] \textbf{R}_{t _i,\ell _i}=(\textbf{B}_{t _i,\ell _i}|\textbf{A})\textbf{R}_{t _i,\ell _i}=\textbf{D}, \end{aligned}$$

as expected. Therefore, the distribution $\textsf{KeyGen}'$-oracle’s replies is, by Lemma 9, statistically close to that of $\textsf{Game}_{5}$.

$\textsf{Game}_{7}$. This is the same as previous game, except for the following modifications. We modify how $\textsf{Enc}'$- and $\textsf{HonUpdate}$-oracles are handled for ciphertexts different from the challenge one. Every time the adversary makes a query to the $\textsf{Enc}'$-oracle of the form $(\vec {x}, t)$, we return $({\textsf{ct}}_{t,1,1},{\textsf{ct}}_{t,1,2})\leftarrow \textsf{Enc} (mpk,t,\vec {x})$, add $(\textsf{c},C _{t},t, \vec {x})$ to ${\mathcal {C}}$, and increment $\textsf{c}$. Whenever the adversary makes a query to the $\textsf{HonUpdate}$-oracle of the form $(t, t ', i,\cdot )$, we check if $(\cdot ,t,t ',\cdot )$ is in $\mathcal{H}\mathcal{T}$ and if $(i,\cdot ,t,\vec {x})$ is in ${\mathcal {C}}$ for some $\vec {x}\in \mathbb {Z} _q^{m}$. If so, we sample $\vec {r}\leftarrow \mathbb {Z} _q^n$, $\vec {g_1}\leftarrow \mathcal {D}_{\mathbb {Z} ^{2\,m}, \tau }$, $\vec {g_2}\leftarrow \mathcal {D}_{\mathbb {Z} ^{m}, \tau }$, and return $({\textsf{ct}}_{t ',2,1},{\textsf{ct}}_{t ',2,2})$, where

$$\begin{aligned} {\textsf{ct}}_{t ',2,1}:=\textbf{H}_{t ',2}^\top \vec {r}+\vec {g}_1, \qquad {\textsf{ct}}_{t ',2,2}:=\textbf{D}_{t '}^\top \vec {r}+\vec {g}_2+\left\lfloor {\frac{q}{K}}\right\rfloor \cdot \vec {x}, \end{aligned}$$

otherwise we return $\bot$. By the Smudging Lemma 12, since the parameter of the Gaussian distribution from which $\vec {f}_1$ and $\vec {f}_2$ are sampled is superpolynomially bigger than the norm of $\Delta _{t \rightarrow t ',1}^\top \vec {f}$ and $\vec {e}_2+\vec {e}_3+\Delta _{t \rightarrow t ',2}^\top \vec {f}$, we get that

$$\begin{aligned} {\textsf{SD}}\left( \mathcal {D}_{\mathbb {Z} ^n, \tau },\mathcal {D}_{\mathbb {Z}, \tau , \Delta _{t \rightarrow t ',1}^\top \vec {f}}\right) ,{\textsf{SD}}\left( \mathcal {D}_{\mathbb {Z} ^n, \tau },\mathcal {D}_{\mathbb {Z}, \tau , \vec {e}_2+\vec {e}_3+\Delta _{t \rightarrow t ',2}^\top \vec {f}}\right) \le \frac{1}{\lambda ^{\omega (1)}}, \end{aligned}$$

where we used again Lemma 9 to bound the norm of $\Delta _{t \rightarrow t ',1}^\top \vec {f}$ and $\vec {e}_2+\vec {e}_3+\Delta _{t \rightarrow t ',2}^\top \vec {f}$. Therefore, the distribution of $\textsf{Enc}'$- and $\textsf{HonUpdate}$-oracle’s replies is statistically close to that of $\textsf{Game}_{6}$.

$\textsf{Game}_{8}$. The only queries for which we still need the main secret key $\textbf{T}_{\textbf{A}}$ are the $\textsf{HonUpdate}$-oracle queries on input the challenge ciphertext, and the functional secret key queries for the challenge tag $t ^*$ (with $\ell =1$) and the tags $t _{j_k}$ with $\{j_k\}_{k\le Q_{\text {u}}}$ (for $\ell =2$). We now perform a reduction to the security of the ALS [15] encryption scheme. We reduce to the ${\textsf{AD}}$-$\textsf{IND}$ security of ALS. We first obtain from the challenger public keys $\textbf{A}_{\text {ALS}}$, $\textbf{D}_{\text {ALS}}$. Now, equipped with the knowledge of $t ^*$, we define $\textsf{Game}_{8}$ to be the same as $\textsf{Game}_{7}$, except for the following changes:

- The matrix $\textbf{A}$ is replaced with $\textbf{A}_{\text {ALS}}$ instead of being generated with $\textsf{TrapGen}$.
- We sample $\textbf{S}_{t ^*}\leftarrow \{\pm 1\}^{m\times m}$ and $\textbf{Z}_{t ^*}\leftarrow \mathcal {D}_{\mathbb {Z} ^{m\times m},\rho _1}$, program $H_1(t ^*): =\textbf{A}\textbf{S}_{t ^*}$ and set $H_3(t ^*):=\textbf{D}_{t ^*}:=\textbf{D}_{\text {ALS}}+\textbf{A}\textbf{S}_{t ^*}\textbf{Z}_{t ^*}$.
- Similarly, for each $k\in [Q_{\text {u}}]$, we sample $\textbf{S}_{t _{j_k}}\leftarrow \{\pm 1\}^{m\times m}$ and $\textbf{R}_{t _{j_k}},\textbf{Z}_{t _{j_k}}\leftarrow \mathcal {D}_{\mathbb {Z} ^{m\times m},\rho _2}$, program $H_2(t _{j_k}): =\textbf{A}\textbf{S}_{t _{j_k}}$ and set $H_3(t _{j_k}):=\textbf{D}_{t _{j_k}}=\textbf{D}_{\text {ALS}}+\textbf{A}\textbf{R}_{t _{j_k}}+\textbf{A}\textbf{S}_{t _{j_k}}\textbf{Z}_{t _{j_k}}$
- For key queries of the form $(t,\vec {y})$, we forward $\vec {y}$ to the challenger of the AD-IND security of ALS, which replies with $sk _{\vec {y}}=\textbf{Z}_{\text {ALS}}\cdot \vec {y}$, where $\textbf{Z}_{\text {ALS}}$ is the main secret key of the ALS scheme. If $t =t ^*$, we set
  $$\begin{aligned} sk _{t ^*,1, \vec {y}}:= \left( \begin{array}{c} sk _{\vec {y}}\\ \hline \textbf{Z}_{t ^*}\vec {y} \end{array} \right) , \end{aligned}$$
  and using $\textbf{T}_{\textbf{B}_{t ^*,2}}$ we compute $sk _{t ^*,2, \vec {y}}$. If $t =t _{j_k}$ for some $k\in [Q_{\text {u}}]$, then we set
  $$\begin{aligned} sk _{t _{j_k},2, \vec {y}}:= \left( \begin{array}{c} sk _{\vec {y}} + \textbf{R}_{t _{j_k}}\vec {y} \\ \hline \textbf{Z}_{t ^*}\vec {y} \end{array} \right) , \end{aligned}$$
  and using $\textbf{T}_{\textbf{B}_{t _{j_k},1}}$ we compute $sk _{t _{j_k},1, \vec {y}}$. One forwards both to the adversary.
- When the adversary finally submits a challenge $(\vec {x}_0, \vec {x}_1)$, we forward it to the ALS challenger, which replies with ${\textsf{ct}}=({\textsf{ct}}^{\text {ALS}}_1, {\textsf{ct}}^{\text {ALS}}_2)$. We compute
  $$\begin{aligned} {\textsf{ct}}_{t ^*,1}&=({\textsf{ct}}^{\text {ALS}}_1 | (\textbf{S}_{t ^*})^\top \cdot {\textsf{ct}}^{\text {ALS}}_1),\\ {\textsf{ct}}_{t ^*,2}&={\textsf{ct}}^{\text {ALS}}_2 + (\textbf{R}_{t ^*}+\textbf{S}_{t ^*}\textbf{Z}_{t ^*})^\top \cdot {\textsf{ct}}^{\text {ALS}}_1+\textsf{NoiseGen}((\textbf{R}_{t ^*}+\textbf{S}_{t ^*}\textbf{Z}_{t ^*})^\top ,s), \end{aligned}$$
  forward $({\textsf{ct}}_{t ^*,1},{\textsf{ct}}_{t ^*,2})$ back to the adversary. (The properties of the algorithm $\textsf{NoiseGen}$ are recalled in Lemma 13 from Appendix A.2.)^17^
- Whenever the adversary queries the $\textsf{HonUpdate}$ oracle on input the challenge ciphertext $({\textsf{ct}}_{t ^*,1},{\textsf{ct}}_{t ^*,2})$ and target tag $t _{j_k}$, we compute
  $$\begin{aligned} {\textsf{ct}}_{t _{j_k},1}&=({\textsf{ct}}^{\text {ALS}}_1 | (\textbf{S}_{t _{j_k}})^\top \cdot {\textsf{ct}}^{\text {ALS}}_1)+\textbf{H}_{t _{j_k},2}^\top \vec {r}+\vec {g}_1, \\ {\textsf{ct}}_{t _{j_k},2}&={\textsf{ct}}^{\text {ALS}}_2 + (\textbf{R}_{t _{j_k}}+\textbf{S}_{t _{j_k}}\textbf{Z}_{t _{j_k}})^\top \cdot {\textsf{ct}}^{\text {ALS}}_1+\textbf{D}_{t _{j_k}}^\top \vec {r}+\vec {g}_2, \end{aligned}$$
  and forward it to the adversary.

In this game, the advantage of the adversary is upper bounded by the advantage of breaking the ALS scheme, i.e., that $\textbf{Adv}_{\text {Game}_8}(\mathcal {A})\le \textbf{Adv}_{\text {ALS}}(\mathcal {A})$. It remains to show that $\textsf{Game}_{8}$ is indistinguishable from $\textsf{Game}_{7}$. We show that the update of the challenge ciphertext and function keys for tag $t _{j_k}$, with $k\in [Q_{\text {u}}]$, are statistically close to those obtained in $\textsf{Game}_{7}$. An identical argument to that used in [9] proves the same for the challenge tag $t ^*$. We start by considering the function keys. Since the parameter of the Gaussian distribution from which $\textbf{R}_{t _{j_k}}$ is sampled is superpolynomially bigger than the norm of $\textbf{Z}_{\text {ALS}}$, by the Smudging Lemma 12 we have that $sk _{\vec {y}}+\textbf{R}_{t _{j_k}}$ is distributed statistically close to $\mathcal {D}_{\mathbb {Z} ^{m\times m},\rho _2}$. Moreover, we have that

$$\begin{aligned} \begin{aligned} \textbf{H}_{t _{j_k},2}\cdot sk _{t _{j_k},2, \vec {y}}&=(\textbf{A}|\textbf{A}\textbf{S}_{t _{j_k}}) \left( \begin{array}{c} sk _{\vec {y}} + \textbf{R}_{t _{j_k}}\vec {y} \\ \hline \textbf{Z}_{t _{j_k}}\vec {y} \end{array} \right) \\&=\textbf{A}sk _{\vec {y}} + \textbf{A}\textbf{R}_{t _{j_k}}\vec {y} + \textbf{A}\textbf{S}_{t _{j_k}}\textbf{Z}_{t _{j_k}}\vec {y}=\textbf{D}_{t _{j_k}}\vec {y}, \end{aligned} \end{aligned}$$

as expected. As far as the update of the challenge ciphertext is concerned, as before, since the parameter of the distribution from which $\vec {g}_2$ is drawn is superpolynomially bigger than the norm of the other error terms in the expression of ${\textsf{ct}}_{t _{j_k},2}$, again by the Smudging Lemma 12, we obtain that the distribution of the ciphertext so obtained is statistically close to that of $\textsf{Game}_{7}$.

Putting everything together, we obtain that

$$\begin{aligned} \textsf{Adv}^{\mathsf {ind\text {-}cufe\text {-}cpa}}_{\textsf{CUFE},\mathcal {A}}(\lambda ,\mathcal {Y})&\le Q_{\text {h}}\left( {\begin{array}{c}Q_{\text {h}} - 1\\ Q_{\text {t}}\end{array}}\right) \left( {\begin{array}{c}Q_{\text {h}} - Q_{\text {t}}-1\\ Q_{\text {u}}\end{array}}\right) \cdot \textbf{Adv}_{\text {ALS}}(\mathcal {A})+\textsf{negl}(n) \\&\le Q_{\text {h}}^{\,(Q_{\text {t}}+Q_{\text {u}}+1)}\cdot \textbf{Adv}_{\text {ALS}}(\mathcal {A})+\textsf{negl}(\lambda ). \square \end{aligned}$$

## Conclusion

In this work, we proposed ciphertext-updatable functional encryption (CUFE), a variant of functional encryption which allows switching ciphertexts produced with respect to one tag to one under another tag using an update token for this tag pair. We have provided practical motivation for such a primitive and then defined an (adaptive) security notion in the indistinguishability setting for CUFE. We presented two constructions, where the first construction is a generic construction of CUFE for any functionality, which can also be extended to predicates other than the equality testing on tags. This construction is based on indistinguishability obfuscation (iO) and is proven to achieve semi-adaptive security. The second construction is a (plausibly) post-quantum CUFE for the inner-product functionality that relies on standard assumptions from lattices. The lattice-based construction achieves the stronger adaptive security notion, albeit with certain restrictions on the validity of the adversary and bound on the number of oracle queries. We leave several questions as interesting open problems. Firstly, to construct a CUFE scheme that satisfies our adaptive security model without any further restrictions or bound on the number of oracle queries. Secondly, to construct practical CUFE schemes for a richer class of functionalities, e.g., quadratic functions, which can further broaden the scope of application. Thirdly, we consider it an interesting direction to study multi-input as well as multi-client extensions of CUFE similarly as it has been done for IB- and AB-IPFE in [9] and [51], respectively.

## Additional Preliminaries

### Functional Encryption with Adaptive Security

We recall the adaptive variant for the security of functional encryption here.

#### Definition 9

For every functional encryption scheme ${\textsf{FE}}$ for functionality $\mathcal {F}:\mathcal {X}\rightarrow \mathcal {Y}$, every security parameter $\lambda$, every PPT adversary *A*, we define the following experiment:

where $\mathcal {O}_{\textsf{KeyGen}}(\cdot )$ is an oracle that on input $f\in \mathcal {F}$, outputs $\textsf{KeyGen}(msk,f)$. Additionally, if *A* ever calls the oracle $\mathcal {O}_{\textsf{KeyGen}}(\cdot )$ on an input $f\in \mathcal {F}$, the challenge queries $x_0^*$, $x_1^*$ must satisfy $f(x_0^*)=f(x_1^*)$. A functional encryption scheme ${\textsf{FE}}$ is ${\textsf{AD}}$-$\textsf{IND}$ secure if for every PPT adversary *A* the advantage function

$$\begin{aligned} \textsf{Adv}^{\mathsf {ind-cpa}}_{{\textsf{FE}},A}(\lambda ,\mathcal {F}):= \left| \Pr \left[ {\textsf{Exp}^{\mathsf {ind-cpa}}_{{\textsf{FE}},A}(\lambda ,\mathcal {F})=1}\right] -1/2\right| , \end{aligned}$$

is negligible in $\lambda$.

### Lattice Preliminaries

#### Definition 10

([54] Learning with errors) Let *q* be a prime, $\chi$ be a public distribution over $\mathbb {Z} _q$ and $\vec {s}$ be uniformly random over $\mathbb {Z} _q^n$. Moreover, $\vec {s}$ is constant across calls to oracles $\mathcal {O}_{\vec {s}}$, or $\mathcal {O}_\$$, defined below:

- Oracle $\mathcal {O}_{\vec {s}}$ outputs samples $(\vec {a}, \langle \vec {a}, \vec {s} \rangle + e)$ where $\vec {a}\leftarrow \mathbb {Z} _q^n$ and $e\leftarrow \chi$ are fresh and independently sampled,
- Oracle $\mathcal {O}_\$$ outputs uniformly random elements of $\mathbb {Z} _q^n\times \mathbb {Z} _q$.

Define another oracle $\mathcal {O}$, which across all calls, is either $\mathcal {O}_{\vec {s}}$ or $\mathcal {O}_\$$. The learning with errors $\text {LWE}_{q, \chi , n}$ problem is to distinguish with non-negligible probability, given access to oracle $\mathcal {O}$, whether it corresponds to $\mathcal {O}_{\vec {s}}$ or $\mathcal {O}_\$$.

#### Lemma 11

([37, 48] Gaussian Tail Bound) For any *n*-dimensional lattice $\Lambda$, $\vec {c}\in span(\Lambda )$, real $\epsilon \in (0,1)$, and $s\ge \eta _\epsilon (\Lambda )$:

$$\begin{aligned} \Pr _{\vec {x}\leftarrow \mathcal {D}_{\Lambda , s, \vec {c}}}[\Vert \vec {x}-\vec {c}\Vert >s\sqrt{n}]\le \frac{1+\epsilon }{1-\epsilon }\frac{1}{2^n}. \end{aligned}$$

Moreover, for any $\omega (\sqrt{\log n})$ function, there is a negligible $\epsilon (n)$ such that: $\eta _\epsilon (\mathbb {Z})\le \omega (\sqrt{\log n})$. In particular, when sampling integers, we have that for any $\epsilon \in (0,\frac{1}{2})$, any $s\ge \eta _\epsilon (\mathbb {Z})$, and any $t\ge \omega (\sqrt{\log n})$:

$$\begin{aligned} \Pr _{x\leftarrow \mathcal {D}_{\mathbb {Z}, s, c}}[|x-c|>s\cdot t]\le \textsf{negl}(n). \end{aligned}$$

#### Lemma 12

(Smudging Lemma) Let $n\in \mathbb {N}$. For any real $\sigma >\omega (\sqrt{\log n})$, and any $\vec {c}\in \mathbb {Z} ^n$, it holds ${\textsf{SD}}(\mathcal {D}_{\mathbb {Z} ^n, \sigma },\mathcal {D}_{\mathbb {Z} ^n, \sigma , \vec {c}})\le \Vert \vec {c}\Vert /\sigma$.

**Noise Re-randomization**. The following procedure of $\textsf{NoiseGen}(\textbf{R},s)$ for noise re-randomization, was described in [44]. $\textsf{NoiseGen}(\textbf{R},s)$: given a matrix $\textbf{R}\in \mathbb {Z} ^{m\times t}$, and $s\in \mathbb {R}^+$ such that $s^2> s_1(\textbf{R}\textbf{R}^\top )$, it first samples $\vec {e}_1:=\textbf{R}\vec {e}+(s^2\textbf{I}_m-\textbf{R}\textbf{R}^\top )^{\frac{1}{2}}\vec {e}'$, where $\textbf{I}_m\in \mathbb {Z} ^{m\times m}$ denotes the identity matrix, and $\vec {e}\leftarrow \mathcal {D}^t_{\sigma }$, and $\vec {e}'\leftarrow \mathcal {D}^m_{\sqrt{2}\sigma }$ are independent spherical continuous Gaussian noises. Then, it samples $\vec {e}_2\leftarrow \mathcal {D}_{\mathbb {Z} ^m-\vec {e}_1,s\sqrt{2}\sigma }$, and return $\vec {e}_1+\vec {e}_2\in \mathbb {Z} _q^m$. We have the following lemma.

#### Lemma 13

([44] Noise Distribution) Let $\textbf{R}\leftarrow \mathbb {Z} ^{m\times t}$ and $s\ge s_1(\textbf{R})$. The following distributions are statistically close: **Distribution 1**: $\vec {e}\leftarrow \mathcal {D}_{\mathbb {Z} ^{t}, \sigma }$, and $\vec {e}'\leftarrow \textsf{NoiseGen}(\textbf{R},s)$. Output $\textbf{R}\vec {e}+\vec {e}'$. **Distribution 2**: Output $\vec {e}\leftarrow \mathcal {D}_{\mathbb {Z} ^m, 2\,s\sigma }$.

#### Lemma 14

([1] Bounding Norm of a $\{\pm 1\}^{k\times m}$ Matrix) Let $\textbf{R}$ be a matrix chosen uniformly at random from $\{\pm 1\}^{k\times m}$. There exists a universal constant $C'$, for which:

$$\begin{aligned} \Pr \left[ \Vert \textbf{R}\Vert \ge C'\sqrt{k+m}\right] <\frac{1}{e^{k+m}}. \end{aligned}$$

#### Lemma 15

([29] Bounding Spectral Norm of a Gaussian Matrix) Let $\textbf{Z}\in \mathbb {R} ^{n\times m}$ be a sub-Gaussian random matrix with parameter $\rho$. There exists a universal constant *C* such that for any $t\ge 0$, we have $s_1(\textbf{Z})\le C\cdot \rho (\sqrt{n}+\sqrt{m}+t)$ except with probability at most $\frac{2}{e^{\pi t^2}}$.

## Footnotes

1 Unless mentioned otherwise, we will always assume public-key functional encryption.

2 https://research.kudelskisecurity.com/2021/02/02/benchmarking-privacy-preserving-motion-detection/.

3 There is more related work such as [14, 27, 30, 40, 41, 58] as discussed in [9], but those schemes either provide less functionality or weaker security.

4 https://research.aimultiple.com/data-marketplace, https://datarade.ai/platform-categories/personal-data-marketplaces.

5 An honest-but-curious party is assumed to correctly perform the update but will not learn any information about the hidden messages.

6 Recall that in a deterministic encryption scheme there are only two such ciphertexts.

7 The decryption algorithms takes either a non-updated ciphertext or an updated one but not both. We assume that one can retrieve the information on the update status from the ciphertexts efficiently.

8 We require honestly generated ciphertexts as input to $\textsf{HonUpdate}$ which we track in the model. This is reminiscent of Cohen’s work [26] which details the necessities of such a restriction.

9 The program is padded to the size equal to $\max \{|\text {PInit:1}[k_{\textsf{prf},o}]|, |\text {PInit:2}[k_{\textsf{prf},o}^{p^*}]|\}$.

10 The program is padded to the size equal to $\max \{|\text {PKey:1}[k_{\textsf{prf},o},k_{\textsf{prf},u},f,t]|,|\text {PKey:2}[k_{\textsf{prf},o}^{p^*},k_{\textsf{prf},u},f,t,p^*,c_0,c_1,v_f,k_{\textsf{ptde}}']|\}$.

11 The program is padded to the size equal to $\max \{|\text {PUpdate:1}[k_{\textsf{prf},o},k_{\textsf{prf},u},t,t']|, |\text {PUpdate:2}[k_{\textsf{prf},o}^{p^*},k_{\textsf{prf},u},t,t',p^*,c_0,c_1,c_0',c_1',k_{\textsf{ptde}}']|\}$.

12 We assume that it is easy to distinguish between updated and fresh ciphertext. This is without loss of generality as we can simply append a bit to the ciphertexts to achieve this distinguishability.

13 If $c_0' < c_1'$, then $c_0 = c_0', c_1 = c_1'$, otherwise, $c_0 = c_1', c_1 = c_0'$.

14 Here we sort $c_0$ and $c_1$ lexicographically and then we order $c_0'$ and $c_1'$ according to this sort, i.e., if $c_0 \leftarrow \Sigma .\textsf{Enc} (k_{\textsf{ptde}}t,m_b)$, then $c_0' \leftarrow \Sigma .\textsf{Enc} (k_{\textsf{ptde}}',t',m_b)$.

15 We refer the reader to Appendix A.1 for a formal definition.

16 observe that this limitation is only *per tag*

17 Notice that it is possible to rely on the Smudging Lemma here as well. To simplify the proof we use the properties of $\textsf{NoiseGen}$, as done by [9], and directly refer to their security proof.
